# Longitudinal assessment of human antibody binding to hemagglutinin elicited by split-inactivated influenza vaccination over six consecutive seasons

**DOI:** 10.1371/journal.pone.0301157

**Published:** 2024-06-25

**Authors:** Michael A. Carlock, James D. Allen, Hannah B. Hanley, Ted M. Ross

**Affiliations:** 1 Center for Vaccines and Immunology, Athens, GA, United States of America; 2 Department of Infectious Diseases, University of Georgia, Athens, GA, United States of America; 3 Florida Research and Innovation Center, Cleveland Clinic, Port Saint Lucie, FL, United States of America; 4 Department of Infection Biology, Lehner Research Institute, Cleveland Clinic, Cleveland, OH, United States of America; University of South Dakota, UNITED STATES

## Abstract

Participants between the ages of 10–86 years old were vaccinated with split-inactivated influenza vaccine (Fluzone®) in six consecutive influenza seasons from 2016–2017 to 2021–2022. Vaccine effectiveness varies from season to season as a result of both host immune responses as well as evolutionary changes in the influenza virus surface glycoproteins that provide challenges to vaccine manufacturers to produce more effective annual vaccines. Next generation influenza vaccines are in development and may provide protective immune responses against a broader number of influenza viruses and reduce the need for annual vaccination. An improved understanding how current influenza vaccines are influenced by human host immune responses in people of different ages and co-morbidities is necessary for designing the next-generation of ’universal’ or broadly-protective influenza vaccines. Overall, pre-existing immune responses to previous influenza virus exposures, either by past infections or vaccinations, is a critical factor influencing host responses to seasonal influenza vaccination. Participants vaccinated in consecutive seasons had reduced serum hemagglutination-inhibition (HAI) activity against strains included in the vaccine compared to participants that had not been vaccinated in the preceding 1–2 years prior to entering this study. The magnitude and breadth of these antibody responses were also modulated by the age of the participant. Elderly participants over 65 years of age, in general, had lower pre-existing HAI titers each season prior to vaccination with lower post-vaccination titers compared to children or young adults under the age of 35. The administration of higher doses (HD) of the split-inactivated vaccine enhanced the antibody titers in the elderly. This report showcases 6 consecutive years of antibody HAI activity in human subjects receiving seasonal split-inactivated influenza vaccine.

## Introduction

Every year influenza viruses cause serious respiratory illnesses in humans that are linked to approximately 290,000–650,000 global deaths [1, 2]. Furthermore, influenza viruses induce acute disease that result in absences from work and school [3, 4]. The associated cost of medical care and reduction in workplace productivity leads to a large economic burden, which in the U.S. is estimated at ~6.3–25.3 billion USD annually [5]. Vaccination is currently the most effective approach for preventing influenza-associated illnesses [6, 7]. However, influenza is an antigenically variable virus which over time acquires amino acid substitutions in its immunodominant glycoproteins, hemagglutinin (HA) and neuraminidase (NA), to evade host immune pressure [2, 8, 9]. Therefore, vaccine effectiveness can vary from season to season and requires commercial vaccines to be updated frequently [10, 11]. Currently in the U.S., there are three types of influenza virus vaccines licensed for human use including split-inactivated virus (IIV), cold-adapted live attenuated virus (LAIV), and recombinant HA (rHA) protein-based vaccines [5]. Vaccine effectiveness varies based on the type of vaccine, as well as previous influenza virus vaccination or infection exposure history [12, 13]. Age also plays a large role in vaccine effectiveness, since aging is associated with diminished humoral and cellular immune responses that impact antibody production and B-cell activation, as well as the induction of antigen specific T-cells [14, 15].

In this study, participants between the ages of 10–86 years old were vaccinated over 6-consecutive influenza seasons, 2016–2017 to 2021–2022, with either the standard (SD) or high dose (HD) formulation of Fluzone® split-inactivated influenza vaccine (IIV) containing two influenza A virus (IAV) strains, H1N1 and H3N2, and one-to-two influenza B virus (IBV) strains, from the Yamagata- or Victoria-lineages. Serum samples collected at the time of vaccination and 21/28 days later were then assessed for the ability to inhibit viral induced hemagglutination against panels of currently circulating and historical strains of influenza virus to determine the effects of age, immune history, and vaccine composition on the induction of seroconversion and seroprotective antibody titers.

## Materials and methods

### Subjects and vaccines

Eligible volunteers between the ages of 10 and 86 years old (y.o.), who had not been administered an annual seasonal influenza vaccine in that year, were enrolled starting in September of each year from 2016 to 2022. The influenza virus was not known to circulate widely in the community during the study time frame, and the participants were not monitored for influenza virus infection throughout the duration of the study. Volunteers were recruited from multiple sites that included medical facilities in and around Athens, Georgia, USA and were enrolled with written, informed consent. Exclusion criteria included body weight less than 110 pounds, medically documented contradictions to Guillain-Barre syndrome, Alzheimer disease or dementia, allergies to eggs or egg-derived products, any medical treatments resulting in or the diagnosis of an immunocompromising condition, an estimated life expectancy less than 2 years, or concurrent participation in another influenza vaccine research study.

In the 2016–2017 season (UGA1), 148 eligible volunteers participated in the study and the demographic information of these individuals were divided by age, gender, race/ethnicity, and body mass index (BMI) (**Table 1**). During the 2017–2018 season (UGA2), 271 eligible participants were enrolled in the study. In the 2018–2019 season (UGA3), 250 volunteers took part in the study. In the 2019–2020 season (UGA4), 461 eligible participants were enrolled. In the 2020–2021 season (UGA5), 339 eligible participants were enrolled, and in the 2021–2022 season (UGA6), 337 eligible participants were enrolled (**Table 1**). All vaccinations and sample collections in this study occurred between September and March of each season, with most occurring before the end of December. From 2019–2022 there was a group of 243 individuals that took part in 3 consecutive seasons of vaccination and sample collections, and the demographics of those individuals were also divided into age, gender, race/ethnicity, and BMI (**Table 2**).

**Table 1:** Demographics of volunteers.

Season (Cohort ID)	Age Groups	Total (#)	Gender			Race/Ethnicity						Body Mass Index (BMI)			
			Female	Male	Other	White	Black or AA	Hispanic or Latino	Asian	Mixed	Other	< 25	25–30	≥ 30	N/A
**2016–2017 (UGA1)**	ALL	148	61.5%	38.5%	0.0%	75.7%	9.5%	6.1%	5.4%	3.4%	0.0%	42.6%	35.8%	21.6%	0.0%
	18–34	93	43.9%	18.9%	0.0%	45.9%	6.1%	4.1%	4.7%	2.0%	0.0%	37.2%	18.2%	7.4%	0.0%
	35–49	19	8.1%	4.7%	0.0%	8.1%	2.0%	2.0%	0.7%	0.0%	0.0%	2.7%	3.4%	6.8%	0.0%
	50–64	21	6.1%	8.1%	0.0%	12.2%	0.7%	0.0%	0.0%	1.4%	0.0%	1.4%	8.1%	4.7%	0.0%
	65–74	15	3.4%	6.8%	0.0%	9.5%	0.7%	0.0%	0.0%	0.0%	0.0%	1.4%	6.1%	2.7%	0.0%
	*65+ (SD)*	*12*	*2*.*0%*	*6*.*1%*	*0*.*0%*	*7*.*4%*	*0*.*7%*	*0*.*0%*	*0*.*0%*	*0*.*0%*	*0*.*0%*	*0*.*7%*	*5*.*4%*	*2*.*0%*	*0*.*0%*
	*65+ (HD)*	*3*	*1*.*4%*	*0*.*7%*	*0*.*0%*	*2*.*0%*	*0*.*0%*	*0*.*0%*	*0*.*0%*	*0*.*0%*	*0*.*0%*	*0*.*7%*	*0*.*7%*	*0*.*7%*	*0*.*0%*
**2017–2018 (UGA2)**	ALL	271	54.6%	45.4%	0.0%	81.5%	7.0%	5.9%	2.2%	2.6%	0.7%	45.0%	31.0%	24.0%	0.0%
	12–17	72	14.8%	11.8%	0.0%	22.1%	0.7%	1.8%	0.4%	1.1%	0.4%	21.8%	2.2%	2.6%	0.0%
	18–34	97	22.1%	13.7%	0.0%	29.2%	1.8%	2.6%	1.1%	0.7%	0.4%	17.0%	14.8%	4.1%	0.0%
	35–49	34	6.3%	6.3%	0.0%	8.9%	2.2%	0.7%	0.4%	0.4%	0.0%	1.8%	3.0%	7.7%	0.0%
	50–64	31	5.5%	5.9%	0.0%	9.2%	1.1%	0.4%	0.4%	0.4%	0.0%	1.8%	4.1%	5.5%	0.0%
	65–83	37	5.9%	7.7%	0.0%	12.2%	1.1%	0.4%	0.0%	0.0%	0.0%	2.6%	7.0%	4.1%	0.0%
	*65+ (SD)*	*21*	*3*.*7%*	*4*.*1%*	*0*.*0%*	*6*.*3%*	*1*.*1%*	*0*.*4%*	*0*.*0%*	*0*.*0%*	*0*.*0%*	*1*.*5%*	*3*.*7%*	*2*.*6%*	*0*.*0%*
	*65+ (HD)*	*16*	*2*.*2%*	*3*.*7%*	*0*.*0%*	*5*.*9%*	*0*.*0%*	*0*.*0%*	*0*.*0%*	*0*.*0%*	*0*.*0%*	*1*.*1%*	*3*.*3%*	*1*.*5%*	*0*.*0%*
**2018–2019 (UGA3)**	ALL	250	56.4%	43.6%	0.0%	84.4%	4.4%	5.2%	2.8%	2.0%	1.2%	62.8%	21.6%	15.6%	0.0%
	12–17	150	32.4%	27.6%	0.0%	51.6%	2.4%	1.6%	1.6%	1.6%	1.2%	49.6%	6.0%	4.4%	0.0%
	18–34	50	13.2%	6.8%	0.0%	16.4%	0.0%	2.4%	0.8%	0.4%	0.0%	10.4%	7.6%	2.0%	0.0%
	35–49	14	3.6%	2.0%	0.0%	4.0%	0.8%	0.4%	0.4%	0.0%	0.0%	0.8%	1.6%	3.2%	0.0%
	50–64	17	4.0%	2.8%	0.0%	5.6%	0.8%	0.4%	0.0%	0.0%	0.0%	0.4%	1.6%	4.8%	0.0%
	65–80	19	3.2%	4.4%	0.0%	6.8%	0.4%	0.4%	0.0%	0.0%	0.0%	1.6%	4.8%	1.2%	0.0%
	*65+ (SD)*	*11*	*1*.*6%*	*2*.*8%*	*0*.*0%*	*3*.*6%*	*0*.*4%*	*0*.*4%*	*0*.*0%*	*0*.*0%*	*0*.*0%*	*0*.*8%*	*3*.*2%*	*0*.*4%*	*0*.*0%*
	*65+ (HD)*	*8*	*1*.*6%*	*1*.*6%*	*0*.*0%*	*3*.*2%*	*0*.*0%*	*0*.*0%*	*0*.*0%*	*0*.*0%*	*0*.*0%*	*0*.*8%*	*1*.*6%*	*0*.*8%*	*0*.*0%*
**2019–2020 (UGA4)**	ALL	461	59.7%	40.3%	0.0%	84.4%	7.4%	4.3%	1.5%	1.5%	0.9%	37.3%	32.5%	30.2%	0.0%
	11–17	99	12.1%	9.3%	0.0%	18.9%	0.9%	0.7%	0.0%	0.9%	0.2%	15.6%	4.1%	1.7%	0.0%
	18–34	90	13.4%	6.1%	0.0%	14.1%	2.4%	1.7%	0.9%	0.4%	0.0%	7.8%	6.9%	4.8%	0.0%
	35–49	91	12.4%	7.4%	0.0%	16.1%	2.4%	0.9%	0.4%	0.0%	0.0%	4.6%	5.9%	9.3%	0.0%
	50–64	91	10.4%	9.3%	0.0%	17.4%	1.3%	0.4%	0.2%	0.0%	0.4%	4.6%	7.6%	7.6%	0.0%
	65–85	90	11.3%	8.2%	0.0%	18.0%	0.4%	0.7%	0.0%	0.2%	0.2%	4.8%	8.0%	6.7%	0.0%
	*65+ (SD)*	*21*	*2*.*2%*	*2*.*4%*	*0*.*0%*	*3*.*9%*	*0*.*2%*	*0*.*2%*	*0*.*0%*	*0*.*2%*	*0*.*0%*	*0*.*9%*	*3*.*0%*	*0*.*7%*	*0*.*0%*
	*65+ (HD)*	*69*	*9*.*1%*	*5*.*9%*	*0*.*0%*	*14*.*1%*	*0*.*2%*	*0*.*4%*	*0*.*0%*	*0*.*0%*	*0*.*2%*	*3*.*9%*	*5*.*0%*	*6*.*1%*	*0*.*0%*
**2020–2021 (UGA5)**	ALL	339	62.2%	37.8%	0.0%	86.4%	5.0%	5.0%	1.8%	0.9%	0.9%	34.8%	31.9%	33.3%	0.0%
	12–17	69	12.1%	8.3%	0.0%	17.1%	1.2%	0.9%	0.6%	0.3%	0.3%	12.4%	5.3%	2.7%	0.0%
	18–34	58	11.5%	5.6%	0.0%	14.2%	0.6%	1.8%	0.3%	0.3%	0.0%	7.4%	4.1%	5.6%	0.0%
	35–49	61	12.1%	5.9%	0.0%	15.0%	1.5%	1.2%	0.3%	0.0%	0.0%	5.0%	5.9%	7.1%	0.0%
	50–64	68	11.5%	8.6%	0.0%	18.0%	1.5%	0.3%	0.0%	0.0%	0.3%	3.2%	7.4%	9.4%	0.0%
	65–85	83	15.0%	9.4%	0.0%	22.1%	0.3%	0.9%	0.6%	0.3%	0.3%	6.8%	9.1%	8.6%	0.0%
	*65+ (SD)*	*7*	*1*.*5%*	*0*.*6%*	*0*.*0%*	*2*.*1%*	*0*.*0%*	*0*.*0%*	*0*.*0%*	*0*.*0%*	*0*.*0%*	*0*.*9%*	*0*.*6%*	*0*.*6%*	*0*.*0%*
	*65+ (HD)*	*76*	*13*.*6%*	*8*.*8%*	*0*.*0%*	*20*.*1%*	*0*.*3%*	*0*.*9%*	*0*.*6%*	*0*.*3%*	*0*.*3%*	*5*.*9%*	*8*.*6%*	*8*.*0%*	*0*.*0%*
**2021–2022 (UGA6)**	ALL	337	58.5%	40.9%	0.6%	85.8%	5.0%	5.0%	1.5%	1.8%	0.9%	38.9%	29.4%	30.0%	1.8%
	10–17	89	12.8%	13.1%	0.6%	20.5%	2.1%	2.1%	0.3%	1.5%	0.0%	18.1%	5.6%	2.7%	0.0%
	18–34	51	10.4%	4.7%	0.0%	13.4%	0.6%	0.9%	0.3%	0.0%	0.0%	5.9%	4.5%	4.5%	0.3%
	35–49	58	10.1%	7.1%	0.0%	14.5%	1.2%	1.2%	0.3%	0.0%	0.0%	4.7%	4.7%	6.5%	1.2%
	50–64	58	10.4%	6.8%	0.0%	15.4%	0.9%	0.3%	0.0%	0.0%	0.6%	2.4%	6.8%	7.7%	0.3%
	65–86	81	14.8%	9.2%	0.0%	22.0%	0.3%	0.6%	0.6%	0.3%	0.3%	7.7%	7.7%	8.6%	0.0%
	*65+ (SD)*	*6*	*1*.*5%*	*0*.*3%*	*0*.*0%*	*1*.*8%*	*0*.*0%*	*0*.*0%*	*0*.*0%*	*0*.*0%*	*0*.*0%*	*0*.*9%*	*0*.*3%*	*0*.*6%*	*0*.*0%*
	*65+ (HD)*	*75*	*13*.*4%*	*8*.*9%*	*0*.*0%*	*20*.*2%*	*0*.*3%*	*0*.*6%*	*0*.*6%*	*0*.*3%*	*0*.*3%*	*6*.*8%*	*7*.*4%*	*8*.*0%*	*0*.*0%*

SD = Standard Dose, HD = High-Dose

AA = African American; Other for Race/Ethnicity = American Indian, Alaskan Native, Native Hawaiian, Pacific Islander, or Semitic

N/A = participants refused to be measured for weight and height

**Table 2:** Demographics of repeater volunteers for three consecutive seasons.

**2019–2022 (UGA 4–6) Repeaters**	**Age Groups**	**Total (#)**	**Gender**			**Race/Ethnicity**						**Body Mass Index (BMI)**		
			**Female**	**Male**	**Other**	**White**	**Black or AA**	**Hispanic or Latino**	**Asian**	**Mixed**	**Other**	**< 25**	**25–30**	**≥s 30**
	ALL	243	62.1%	37.0%	0.8%	89.3%	3.3%	3.7%	0.8%	2.1%	0.8%	32.1%	31.7%	36.2%
	11–17	39	8.6%	6.6%	0.8%	14.4%	0.4%	0.4%	0.0%	0.8%	0.0%	11.5%	2.5%	2.1%
	18–34	35	11.1%	3.3%	0.0%	12.8%	0.4%	0.8%	0.0%	0.4%	0.0%	4.9%	4.9%	4.5%
	35–49	45	11.9%	6.6%	0.0%	15.2%	1.6%	1.2%	0.4%	0.0%	0.0%	5.3%	4.9%	8.2%
	50–64	51	12.3%	8.6%	0.0%	19.3%	0.8%	0.4%	0.0%	0.0%	0.4%	3.3%	8.2%	9.5%
	* 65–86	73	18.1%	11.9%	0.0%	27.6%	0.0%	0.8%	0.4%	0.8%	0.4%	7.0%	11.1%	11.9%

* 52 (71.2%) subjects received HD each season, 6 (8.2%) received SD each season, and 15 (20.5%) swapped from SD to HD: 11 (15.1%) after UGA4, 4 (5.5%) after UGA5.

All study participants were vaccinated intramuscularly (IM) with Fluzone^®^ (Sanofi Pasteur, Swiftwater, PA, USA), an inactivated influenza vaccine (IIV) derived from embryonated chicken eggs. All individuals were immunized with a quadrivalent standard-dose vaccine (QIV SD) containing 15μg/component, and individuals aged 65 or older were offered a choice of being vaccinated with either QIV SD or a high-dose formulation containing 60μg/component (**Table 3**). For UGA1-4, this was a trivalent high-dose (TIV HD) formulation with only one influenza B strain, and for UGA5-6 this was a quadrivalent high-dose (QIV HD) formulation matching the four strains in the QIV SD (**Table 3**).

**Table 3:** Fluzone vaccine formulations for northern hemisphere seasons 2016–2017 to 2021–2022.

Year	Influenza Season	Formulation	IAV (H1N1)	IAV (H3N2)	IBV (Victoria)	IBV (Yamagata)
**UGA1**	2016–2017	QIV SD TIV HD	A/California/07/2009	A/Hong Kong/4801/2014	B/Brisbane/60/2008	* B/Phuket/3073/2013
**UGA2**	2017–2018	QIV SD TIV HD	A/Michigan/45/2015	A/Hong Kong/4801/2014	B/Brisbane/60/2008	* B/Phuket/3073/2013
**UGA3**	2018–2019	QIV SD TIV HD	A/Michigan/45/2015	A/Singapore/INFIMH-16-0019/2016	**^** B/Colorado/6/2017	* B/Phuket/3073/2013
**UGA4**	2019–2020	QIV SD TIV HD	A/Brisbane/02/2018	A/Kansas/14/2017	**^** B/Colorado/6/2017	* B/Phuket/3073/2013
**UGA5**	2020–2021	QIV SD QIV HD	A/Guangdong-Maonan/SWL1536/2019	A/Hong Kong/2671/2019	B/Washington/02/2019	B/Phuket/3073/2013
**UGA6**	2021–2022	QIV SD QIV HD	A/Victoria/2570/2019	**^** A/Tasmania/503/2020	B/Washington/02/2019	B/Phuket/3073/2013

* The high-dose (HD) formulation was a trivalent influenza vaccine (TIV) prior to the 2020–2021 season; for UGA1-UGA4, the TIV formulation did not include the B/Yamagata component

^ Vaccine formulation included B/Maryland/15/2016 (a B/Colorado/6/2017-like strain), but B/Colorado/6/2017 virus used for all assays; A/Tasmania/503/2020 = A/Cambodia/e0826360/2020-like strain

The trial was approved by multiple institutional review boards (Western Institutional Review Board and The University of Georgia Review Board) and all subjects provided written informed consent at the time of enrollment. Data was accessed to generate figures on September 23, 2023. All quadrivalent vaccine formulations consisted of four strains of influenza virus specified by the U.S. Food and Drug Administration for inclusion in the annual vaccine for that year. During the UGA1 (2016–2017) season, the four viral strains included in the quadrivalent formulation were A/California/07/2009 (H1N1), A/Hong Kong/4801/2014 (H3N2), B/Brisbane/60/2008 (Victoria lineage; VIC), and B/Phuket/3073/2013 (Yamagata lineage; YAM), and the three strains in the trivalent formulation were A/California/07/2009 (H1N1), A/Hong Kong/4801/2014 (H3N2), B/Brisbane/60/2008 (VIC) (**Table 3**). In the UGA2 season (2017–2018), the four strains included in the quadrivalent vaccine were A/Michigan/45/2015 (H1N1), A/Hong Kong/4801/2014 (H3N2), B/Brisbane/60/2008 (VIC), and B/Phuket/3073/2013 (YAM), and the three strains included in the trivalent formulation were A/Michigan/45/2015 (H1N1), A/Hong Kong/4801/2014 (H3N2), B/Brisbane/60/2008 (VIC). In the UGA3 season (2018–2019), the four influenza isolates included in the quadrivalent formulation were A/Michigan/45/2015 (H1N1), A/Singapore/INFIMH-16-00119/2016 (H3N2), B/Maryland/15/2016 (VIC), and B/Phuket/3073/2013 (YAM), while the trivalent formulation of the vaccine contained A/Michigan/45/2015 (H1N1), A/Singapore/INFIMH-16-00119/2016 (H3N2), B/Maryland/15/2016 (VIC). For UGA4, the four strains included in the quadrivalent vaccine for both the standard and high dose formulations were A/Brisbane/02/2018 (H1N1), A/Kansas/14/2017 (H3N2), B/Maryland/15/2016 (VIC), and B/Phuket/3073/2013 (YAM). For UGA5, the four strains included in the quadrivalent vaccine for both the standard and high dose formulations were A/Guangdong-Maonan/SWL1536/2019 (H1N1), A/Hong Kong/2671/2019 (H3N2), B/Washington/02/2019 (VIC), and B/Phuket/3073/2013 (YAM). For UGA6, the four strains included in the quadrivalent vaccine for both the standard and high dose formulations were A/Victoria/2570/2019 (H1N1), A/Tasmania/503/2020 (H3N2), B/Washington/02/2019 (VIC), and B/Phuket/3073/2013 (YAM) (**Table 3**).

Blood (70-90mL) was collected from each participant at the time of vaccination (D0), 7–9 days later (D7), and 21–28 days (D28) post-vaccination. Blood samples were processed for sera and peripheral blood mononuclear cells (PBMC) at all three time points. Sera and PMBC samples were aliquoted within 24 hours of collection. Sera samples were stored at -30°C ± 10°C and PBMC samples were stored in liquid nitrogen at -150°C ± 10°C for future analysis. In this study, sera collected at D0 and D28 were analyzed for antibodies capable of mediating hemagglutination inhibition (HAI) against panels of H1N1, H3N2, and influenza B viruses representing historical and current WHO selected influenza vaccine strains.

### Hemagglutination inhibition (HAI) assay

The hemagglutination inhibition assay was used to assess anti-HA directed antibodies that prevent the agglutination of avian red blood cells (RBCs) by H1N1, H3N2, and influenza B viruses. The protocols used for this assay were adapted from the World Health Organization (WHO) laboratory influenza virological surveillance manual [16]. Sera samples were treated with receptor destroying enzyme (RDE) (Denka, Seiken, Co., Japan) to inactivate nonspecific inhibitors, according to the manufacturer’s instructions prior to their use in the assay. In brief, three volumes of RDE were added to one volume of sera and incubated overnight at 37°C. The next day, the RDE was inactivated by incubating the samples at 56°C in a water bath for 30–60 minutes, after which 6 volumes of 1x phosphate buffered saline (PBS) were added to each sample, resulting in a final serum dilution of 10. RDE treated sera was then diluted in a series of two-fold serial dilutions in 96-well V-bottom plates (Thermo Fisher, Waltham, MA, USA), and an equal volume of influenza virus, adjusted to 8 hemagglutination units (HAU)/50μL diluted in 1xPBS, was added to each well of the plate. The plates were then covered and allowed to incubate at room temperature for 20 minutes. After incubation, 50μL of a solution consisting of 0.8% turkey RBCs (Lampire Biologicals, Pipersville, PA, USA) diluted in 1xPBS was added to each well. The plates were then mixed by gentle agitation, covered, and allowed to incubate for another 30 minutes at room temperature. Prior to use, the turkey RBCs were washed twice with 1xPBS, stored at 4°C and used within 24 h of preparation. After incubation with RBCs the plates were tilted to observe the hemagglutination inhibition. The HAI antibody titer was determined by taking the reciprocal dilution of the last well that contained non-agglutinated RBCs. Positive control serum from previously performed mouse or ferret infections were also included to confirm assay consistency between runs. For this study, a “seropositive” HAI reaction is defined as any HAI titer ≥40 and “seroconversion” is defined as a post-vaccination seropositive HAI titer with a 4-fold increase, as per the European Committee for Proprietary Medicinal Products (CPMP) and Food and Drug Administration (FDA) guidelines for evaluating influenza vaccines [17, 18]. “Seroprotection” is used to define any post-vaccination HAI titer ≥40, and “seronegative” is defined as an HAI titer <40.

### Viruses

The influenza viruses used in this study were obtained either through the Influenza Reagents Resource (IRR), BEI Resources (BEI), or were provided by Sanofi Pasteur. To better match the egg-derived Fluzone^®^ (Sanofi Pasteur, Swiftwater, PA, USA) vaccinations, all viruses used in this study were propagated in eggs. Virus stocks were passaged once in 10-day old embryonated chicken eggs as per the instructions provided by the World Health Organization [16]. All virus preparations were titrated with turkey red blood cells, made into single use aliquots, and stored at -80°C. The H1N1 viruses used in this study included: A/California/07/2009 (CA/09), A/Michigan/45/2015 (Mich/15), A/Brisbane/02/2018 (Bris/18), A/Guangdong-Maonan/SWL1536/2019 (GD/19), and A/Victoria/2570/2019 (Vic/19). The H3N2 viruses used in this study included: A/Hong Kong/4801/2014 (HK/14), A/Singapore/INFIMH-16-0019/2016 (Sing/16), A/Kansas/14/2017 (KS/17), A/South Australia/34/2019 (SA/19), A/Hong Kong/2671/2019 (HK/19), A/Tasmania/503/2020 (Tas/20), and A/Darwin/9/2021 (Dar/21). The Yamagata-lineage influenza B virus was B/Phuket/3073/2013 (B/Phu/13). The Victoria-lineage influenza B viruses used in this study included the following representatives: B/Brisbane/60/2008 (B/Bris/08), B/Colorado/06/2017 (B/CO/17), and B/Washington/02/2019 (B/WA/19). All influenza B viruses used in HAI assays are subsequently treated with anhydrous, diethyl ether.

### Statistical methods

Statistical significance of the HAI data was calculated using a two-tailed, paired, non-parametric Students t-test with Wilcoxon matched-pairs signed rank comparing day 0 to day 28 using GraphPad Prism software (GraphPad, San Diego, CA, USA). For this study, a p value ≤0.05 was defined as statistically significant (* = p ≤0.05, ** = p ≤0.01, *** = p ≤0.001, **** = p ≤0.0001).

## Results

### Demographics of volunteers

During the 2016–2017 (UGA1) collection season, 148 volunteers participated in the study. This cohort was predominately female (~62%), white (~76%) and aged 18–34 y.o. (~63%). The UGA1 cohort also had the largest participation from African Americans (~10%) and Hispanic (6%) out of all the UGA1-6 cohorts (**Table 1**). In the following season, 2017–2018, the UGA2 cohort had 271 participants and was also majority female (~55%), white (82%), BMI <25 (~45%), and aged 18–34 y.o. (~36%). The UGA2 season was the first cohort where adolescents aged under 18 y.o. were enrolled in the study and they represented ~26% of the enrolled participants. The UGA2 cohort also had the largest male participation (~45%) out of all six seasons (**Table 1**). The UGA3 collection season, 2018–2019, had 250 enrolled participants that were predominantly female (~56%), white (84%), had an average BMI <25 (~63%), and included the largest participation from individuals aged 12–17 y.o. (~60%) in any of the seasons analyzed (**Table 1**). The UGA4 season, 2019–2020, saw the largest number of enrollees out of all 6 seasons with 461 participants. This cohort was majority female (60%), white (~84%), had an average BMI <25 (~37%), and was predominately aged 11–17 y.o. (~20%). This season also had the largest percentage of individuals with BMIs between 25–30 (~32%) (**Table 1**). The UGA5 cohort, 2020–2021, had 339 participants, was predominantly female (~62%), white (~86%), BMI <25 (35%), and over 65 y.o. (~25%). This cohort also had the highest percentage of individuals with BMI ≥30 (~33%) and had the highest percentage of individuals ≥50 y.o. (~45%) (**Table 1**). The UGA6 cohort, 2021–2022, had 337 enrolled participants and was majority female (~59%), white (86%), had an average BMI <25 (39%), and was aged 10–17 y.o. (~26%) (**Table 1**).

From these seasons, a longitudinal cohort of 243 individuals participated in the UGA4-6, were analyzed over 3 consecutive influenza seasons (**Table 2**). This longitudinal cohort was predominantly female (~62%), white (~89%), with an average BMI ≥30 (~36%) representing each of the 5 age categories. The antigenic composition of the Fluzone® vaccine changed each season (**Table 3**). The H1N1 component changed all 3 years, first from BR/18 to GD/19 in 2020–2021 and then to Vic/19 in the 2021–2022 season. The H3N2 component also changed in all 3 seasons, first from KS/17 to HK/19 in 2020–2021, and then to Tas/20 in 2021–2022. The B/Victoria component was B/CO/17 in 2019–2020, changed to B/WA/19 in the 2020–2021 season, and remained as B/WA/19 in the 2021–2022 season. The B/Yamagata antigenic component remained the same, B/Phu/13, for all 3 seasons from 2019–2022 (**Table 3**).

### Hemagglutination inhibition (HAI) specific antibody responses to vaccination

**Fig 1:**
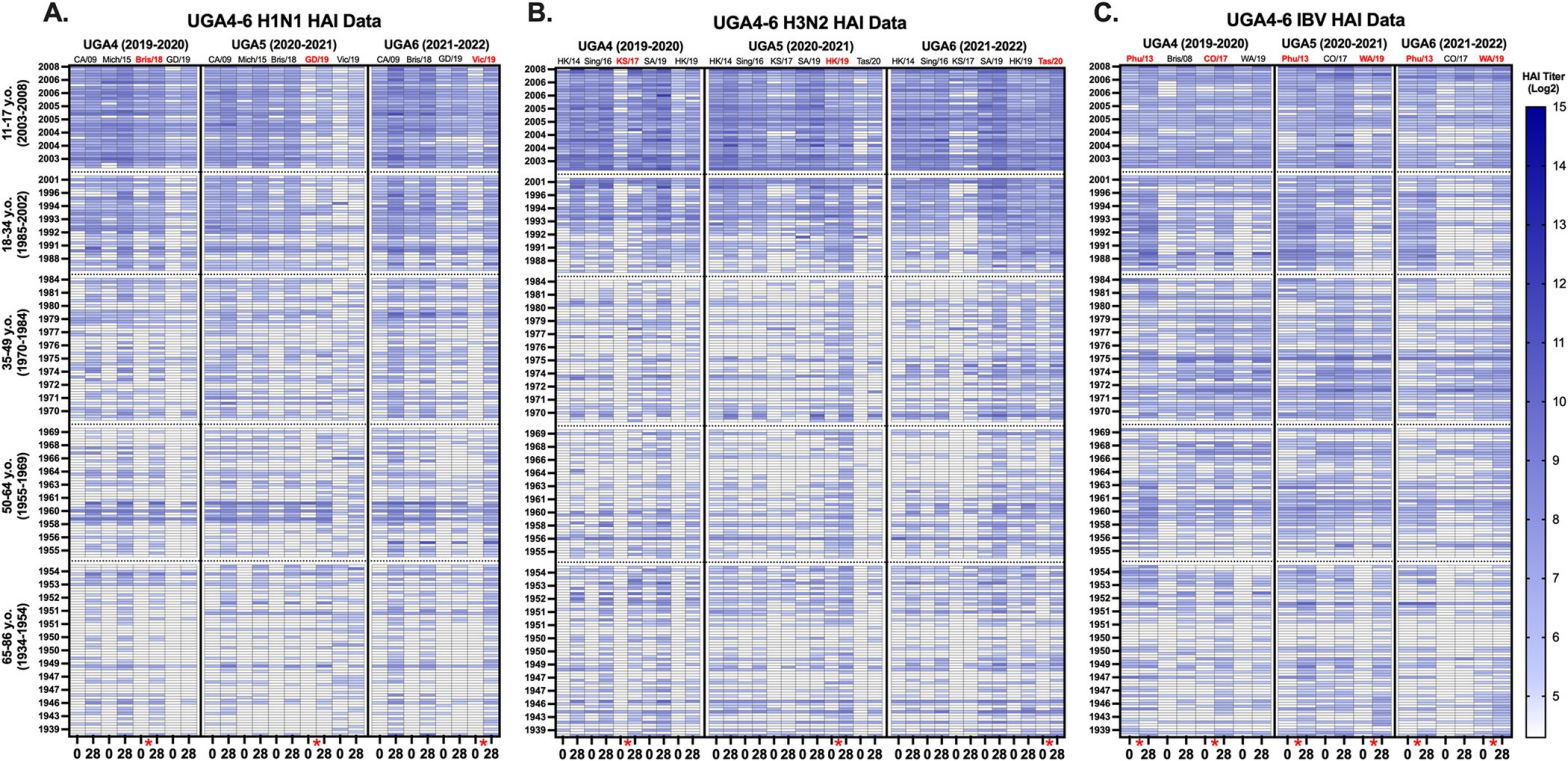
Heat map of HAI activity. Serum samples were collected from subjects at day 0 and day 28 post-vaccination and HAI activity of each serum samples was tested against a panel of IAV and IBV historical influenza viruses. A heat map of each HAI titer against each strain is depicted for UGA4 (2019–2020), UGA5 (2020–2021), and UGA6 (2021–2022) for (A) A/H1N1, (B) A/H3N2, (C) B/Yamagata, and B/Victoria strains. The list of strains is shown on the upper x-axis in chronological order from left to right, oldest to youngest. The subjects were categorized by each age group (youngest to oldest listed from top to bottom of the heat map) on the y-axis. HAI titers (Log2) greater than 40 are highlighted on a blue color scale with lighter color indicating lower HAI titers with darker color for higher HAI titers. HAI titers less than 40 are not colored (20, 10, and 5).

Serum samples collected from individuals on the day of vaccination (D0) and 21–28 days later (D28) were assessed for the presence of antibodies that bind to historical and contemporary H1N1, H3N2, and influenza B vaccine strain viruses and prevent the agglutination of turkey red blood cells *in vitro*, via the hemagglutination inhibition assay (HAI). In the UGA4 cohort, individuals aged 11–17 y.o. had the highest HAI reactive antibody titers at D0 against the H1N1 viruses from 2009–2019 compared to the other age groups (**Fig 1A**). Following vaccination, nearly all the individuals in this age group had seroprotective HAI antibody titers against the viruses in the H1N1 panel, with their highest responses directed against the Mich/15 and Bris/18 viruses. The seroprotective antibody titers against these viruses were also maintained into D0 of the following year for the viruses from 2009–2018. Most of the individuals in this age group enrolled in UGA4 also had seroprotective antibodies against the GD/19 isolate. However, most people did not maintain these titers to day 365 or D0 of the following season. Once vaccinated with the GD/19 strain in the UGA5 season, this group had seroprotective levels of antibodies against the GD/19 strain that were then maintained to the following season (**Fig 1A**). The 18–34 y.o. participants had similar HAI responses as the 11–17 y.o. group following vaccination, but this cohort had fewer participants with seroprotective antibodies at D0 of each season compared to the 11–17 y.o. group (**Fig 1A**). Nearly all the participants in this group had seroprotective antibody responses following vaccination against the vaccine strains for each season, with observed back-boosting to H1N1 strains from 2009–2018, but less seroprotective antibody titers at D0 against the recently circulating strains in each season compared to the younger groups (**Fig 1A**). The vaccine in each season did induce seroprotective antibodies against the panel of viruses in some participants in these groups at D28 post-vaccination, but in general, as people age, these responses diminished and the back-boosting effect induced by the vaccine also declined (**Fig 1A**).

The antibody responses in the same individuals were also analyzed against a panel of historical H3N2 vaccine strains from 2014–2019 (**Fig 1B**). Similar to the results from the H1N1 analysis, most of the individuals aged 11–17 y.o. entered the 2019–2020 season with seroprotective antibody titers at D0 against most of the contemporary H3N2 viruses in the panel (**Fig 1B**). The lowest antibody titers were observed against the KS/17 isolate which was included in the vaccine formulation during the 2019–2020 season. Following vaccination with the KS/17 antigen, nearly all the individuals in this age group had seroprotective HAI antibody titers against the KS/17 virus and those titers were maintained at protective levels into D0 of the subsequent seasons (**Fig 1B**). In comparison, there were fewer 18–34 y.o. individuals in the UGA4 season that had seroprotective antibody titers at D0 across the panel of viruses compared to the younger age group, and like the 11–17 y.o. group, the lowest antibody titers at D0 were observed against the KS/17 strain. Following vaccination, the KS/17 component of the vaccine generated seroprotective antibody levels in most of the individuals in this age group, but in fewer individuals than what was observed in the 11–17 y.o. cohort (**Fig 1B**). However, unlike the younger age group, in the following seasons antibody titers against KS/17 began to wane in this age group, and these responses were not boosted following vaccination with HK/19 and Tas/20 in 2020–2021 and 2021–2022, respectively. In the UGA5 and UGA6 seasons the 18–34 y.o. individuals generated seroprotective antibody responses to the vaccine antigens and in general, back-boosting to historical strains was observed for all viruses, with the exception of KS/17 (**Fig 1B**). In comparison, there were fewer individuals aged ≥35 y.o. that entered each season at D0 with seroprotective antibodies against the panel of H3N2 viruses (**Fig 1B**). Following vaccination, these individuals were seroprotected against the vaccine strain virus from each season, but less back-boosting to historical H3N2 strains was observed across the panel of H3N2 viruses compared to the younger age groups (**Fig 1B**).

The HAI reactive antibody responses were also analyzed against a panel of influenza B viruses for the UGA4-6 repeater cohort (**Fig 1C**). In general, individuals aged 11–17 y.o. entered the UGA4 season with seroprotective HAI antibodies against influenza B viruses from both Yamagata (B/Phu/13) and Victoria (B/Bris/08, B/CO/17, and B/WA/19) lineages. These titers were maintained season to season against the B/Phu/13 virus and against the Victoria lineage component of the vaccine from the previous seasons in UGA5 and UGA6 (**Fig 1C**). In general, following vaccination, the 11–17 y.o. individuals had seroprotective responses against the viruses included in the vaccine formulation and elicited seroprotective back-boosting responses to the historical influenza B strains in the panel (**Fig 1C**). Similar trends were observed in the 18–34 y.o. cohort, where most individuals entered the UGA4 season with seroprotective antibody titers against the B/Phu/13 virus and these responses were maintained following annual vaccination from one season to the next. There were fewer individuals with seroprotective antibody titers at D0 in each season against the Victoria-lineage viruses, B/CO/17 and B/WA/19, however, vaccination generally induced seroprotective antibody titers in each season (**Fig 1C**). In contrast, fewer individuals aged ≥35 y.o. entered the UGA4 season with seroprotective antibody titers against the B/Phu/13 isolate. The vaccine did induce seroprotective antibodies against the viruses matched to the antigens included in the vaccines in most individuals aged ≥35 y.o. for each season, but these titers were not maintained into the following seasons (**Fig 1C**).

### Average HAI titers by season

**Fig 2:**
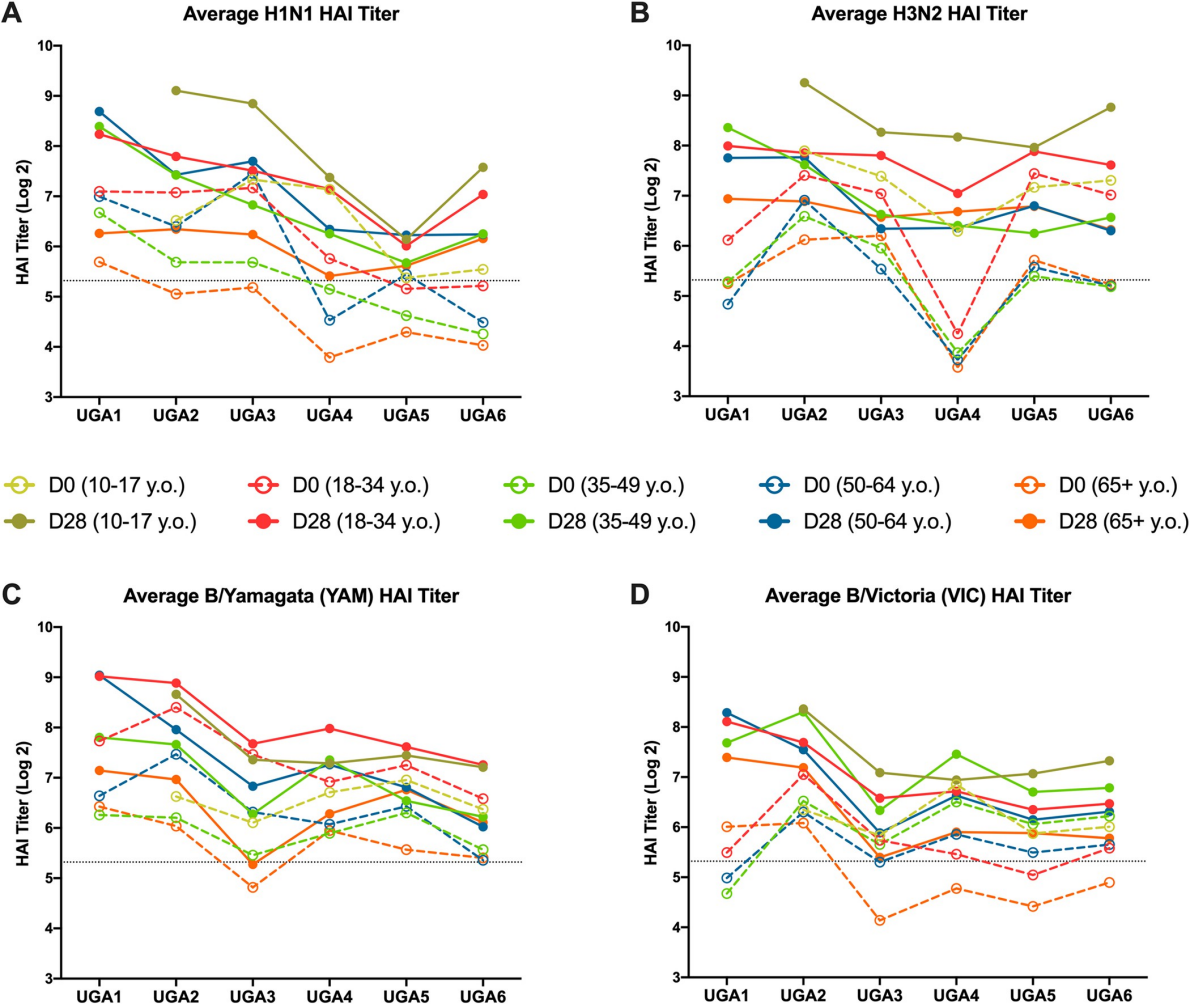
Longitudinal HAI titer. The average HAI activity from serum samples collected from subjects at day 0 and day 28 post-vaccination were assayed against each vaccine component per seasonal vaccine in UGA1 through UGA6 cohorts. The average titer is recorded per each of the 5 age groups for day 0 (open circle, dotted line) and day 28 (closed circle, solid line) per UGA cohort per line graph. Serum sample collected from participants 10–17 y.o. (gold), participants 18–34 y.o. (red), participants 35–49 y.o. (green), participants 50–64 y.o. (blue), participants 65–86 y.o. (orange). Average HAI titer for H1N1 vaccine components (Panel A), average HAI titer for H3N2 vaccine components (Panel B), average HAI titer for B/Yamagata vaccine components (Panel C), average HAI titer for B/Victoria vaccine components (Panel D).

The average HAI titers for each age group against the individual H1N1, H3N2, and influenza B vaccine strains from each season were also analyzed across the UGA1-6 cohorts **(Fig 2**). In general, the vaccine used in each season induced a significant rise in antibody titers from D0 to D28 in every age group against the viruses matched to the vaccine antigens (**S1–S6 Figs**). Individuals aged 10–17 y.o. entered each season with the highest average antibody titers at D0 and generated the highest average antibody titers at D28 against the H1N1 component in each vaccine compared to the other age groups **(Fig 2A**). In this age group, the UGA5 cohort had the lowest average post-vaccination HAI titer against the H1N1 vaccine component, GD/19, and in general that trend was true for all the other age groups **(Fig 2A**). The UGA1 cohort had the highest average HAI response to the H1N1 component of the vaccine and the 50–64 y.o. group had the highest antibody titers during this season **(Fig 2A**). Participants over 65 y.o. entered each season with the lowest antibody titers to the H1N1 component of the vaccine and had the lowest average HAI titers following vaccination **(Fig 2A**).

Across the six seasons (UGA1-6), the highest average HAI reactive H3N2 antibody titers at D0 and D28 were detected in the 10–17 y.o. age group **(Fig 2B**). In general, the vaccine from each season elicited a D28 seroprotective antibody response against the H3N2 component of the vaccines in all age groups. The UGA4 cohort had the lowest antibody titers at D0 to the vaccine strains from that season, KS/17, compared to the other seasons, but on average, following vaccination, all the age groups elicited seroprotective antibody titers against the KS/17 virus **(Fig 2B**). The oldest age group, 65+ y.o., tended to have the lowest antibody titers on D0 and D28 against the H3N2 component of the vaccine from each season **(Fig 2B**).

Participants less than 35 y.o. had the highest average serum HAI activity against the B/Yamagata component of the vaccine across UGA1-6, and the participants over 65 y.o. had the lowest average antibody titers **(Fig 2C**). In general, the antibody titers elicited to the B/Yamagata component of the vaccine declined from 2016–2022, but in each season the vaccine induced seroprotective antibody titers at D28 in all the age groups. The highest average antibody response was elicited by the 2016–2017 vaccine and the lowest by the 2021–2022 vaccine **(Fig 2C**). On average, the annual vaccine in each season from 2016–2022 also elicited seroprotective antibody titers against the B/Victoria component in every age group following vaccination **(Fig 2D**). The highest average antibody responses were observed in the 10–17 y.o. group and the lowest were observed in the ≥65 y.o. age group, which struggled to maintain protective antibody titers at D0 of each year following the 2017–2018 season (UGA2), but this group achieved seroprotective antibody titers at D28 following vaccination each season **(Fig 2D**).

### Seroconversion and seroprotective antibody titers

**Fig 3:**
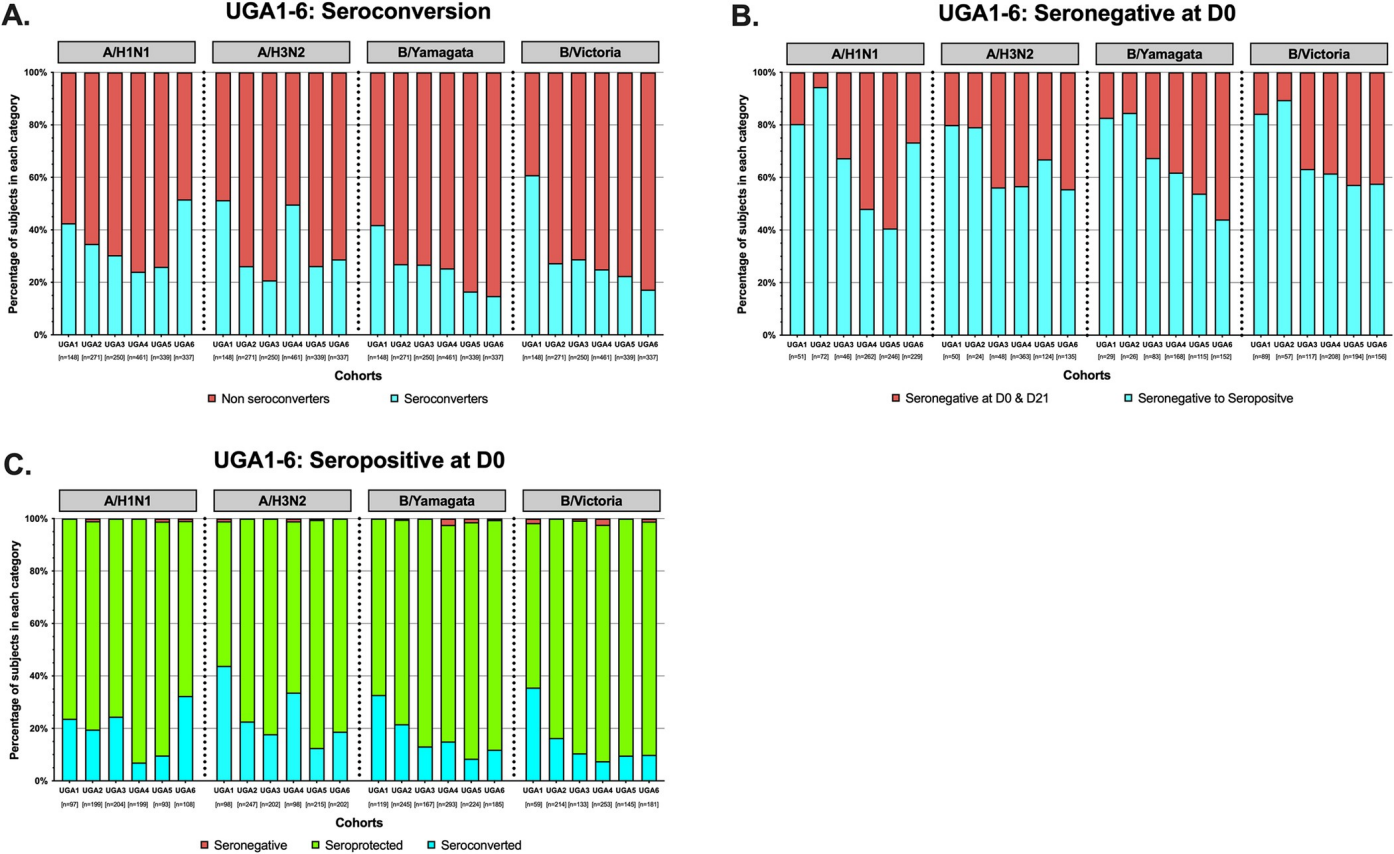
Seroprotection vs. seroconversion. Stacked bar graphs represent the seroconversion to the A/H1N1, A/H3N2, B/Yamagata, and B/Victoria components per season. The number of participants for each cohort are listed on the x-axis. (A) participants were determined to be seroconverters following a 4-fold rise in HAI titer with a titer ≥40 at day 28 (blue) or non-seroconverters (red). (B) Participants seronegative (<40) at day 0 prior to vaccination that seroconverted (four-fold rise in titer with a titer of ≥40) at day 28 are highlighted in blue and those subjects that remained seronegative (<40) at both day 0 and 28 are highlighted in red. (C) Participants that were seropositive (≥40) at day 0 prior to vaccination that seroconverted (4-fold rise in titer) at day 28 are highlighted in blue and those subjects that dropped in titer to a seronegative state (<40) are highlighted in red. Subjects that were seropositive at day 0 and remained seropositive at day 28 are highlighted in green.

The serum HAI activity following vaccination of participants in UGA 1–6 was also assessed by analyzing rates of seroconversion (four-fold rise in titer from D0), seronegative (HAI titer <40) and seropositive (HAI titer ≥40) (**Fig 3**). These categories were based on HAI activity against the vaccine strain for the H1N1, H3N2, and influenza B components in each season. Across all 6 seasons, the cohort had an average seroconversion rate of ~34% to the H1N1 component of the vaccine (**Fig 3A**). The UGA6 cohort had the highest percentage (~52%) of participants that seroconverted to the H1N1 component of the vaccine, and the UGA4 cohort had the lowest rate of seroconversion (~24%) (**Fig 3A**). The H3N2 components induced a similar rate of seroconversion (~34%) compared to the H1N1 component across all 6 seasons. The UGA1 cohort had the highest rate of seroconversion (~51%) and the UGA3 cohort had the lowest rate of seroconversion (~20%) to the H3N2 components of the vaccine. However, in the following year, the UGA4 cohort had a greater than 2-fold rise in seroconversion rate (~50%) with the introduction of the KS/17 antigen to the vaccine (**Fig 3A**). The lowest seroconversion rate across the 6 seasons was against the B/Yamagata component (~24%), which was ~10% lower than the rate of seroconversion to the H1N1 and H3N2 components. The UGA1 cohort had the highest rate of seroconversion (~42%) and the UGA6 cohort had the lowest percentage of participants that seroconverted (~15%) against the Yamagata components, which was nearly 3 times lower than the UGA1 cohort (**Fig 3A**). Approximately 27% of the participants across the UGA1-6 cohorts seroconverted to the B/Victoria components of the vaccine. The UGA1 cohort had the highest percentage of seroconversion events (~61%) out of any of the seasons and across any of the antigens, and in general this number steadily declined in the following seasons to ~17% in the UGA6 cohort (**Fig 3A**).

The number of participants who entered each season with a seronegative HAI titer at D0 and either stayed seronegative or seroconverted at D28 following vaccination was also analyzed (**Fig 3B**). Across the UGA1-6 seasons, ~59% of the participants who were seronegative at D0 seroconverted at D28 to the H1N1 component of the annual vaccine following vaccination. The UGA2 cohort had the highest percentage of individuals that fell into this category (~94%) and the UGA5 cohort had less than half that number (~41%) which was the lowest of any season. In the following season, UGA6, that number jumped up to ~73%, with the introduction of the Vic/19 strain to the vaccine (**Fig 3B**). On average, across the all UGA1-6 cohorts, ~60% of the participants who were seronegative at D0 had seropositive titers at D28 (**Fig 3B**). The UGA1 cohort had the highest percentage (~80%) of individuals that entered seronegative and seroconverted to the H3N2 component of the vaccine at D28 post vaccination, and the UGA6 cohort had the lowest percentage (~56%) of individuals in this category. On average, across the 6 seasons analyzed, the smallest percentage of participants (~59%) who were seronegative at D0 and seroconverted at D28 were to the B/Yamagata components of vaccine compared to the other vaccine antigens. The UGA2 cohort had the highest percentage of these individuals (~85%), and the UGA6 season had the lowest percentage (~44%) that fell into this category (**Fig 3B**). In contrast, across UGA1-6, the B/Victoria components of the vaccine induced the highest frequency of individuals (~64%) that went from seronegative to seropositive. The UGA2 cohort had the highest percentage of participants that fell into this category (~89%) and the UGA5 cohort had the lowest percentage of these individuals (~57%) (**Fig 3B**).

Participants who entered the study seropositive to each of the antigenic components of the vaccine were also categorized into those who stayed seropositive, those who seroconverted, and those who lost antibody titer and were seronegative 28 days after vaccination (**Fig 3C**). On average, ~81% of the participants who entered each season as seropositive on D0 to the H1N1 component of the vaccine maintained their seropositive status 28 days post vaccination, and ~19% of those participants had a 4-fold rise in titer. The UGA4 cohort had the highest percentage of people (~93%) who had seropositive antibody titers at D0 and were still seropositive at D28, and the UGA6 cohort had the smallest frequency of individuals (~67%) in this category. Conversely, the UGA4 cohort had the smallest percentage of participants (~7%) who were seropositive at D0 and seroconverted at D28 to the H1N1 component of the vaccine, and the UGA6 cohort had the highest frequency (~32%) of these individuals (**Fig 3C**). Across the UGA1-6 cohorts, ~78% of the participants entered each year seropositive on D0 to the H3N2 component of the vaccine and maintained their seropositive status 28 days post vaccination. The UGA1 cohort had the lowest proportion of these participants (~55%) and the UGA5 cohort had the highest percentage of participants (~87%) who fell into this category. Conversely, the UGA1 cohort had the highest percentage of participants (~44%) who seroconverted following vaccination after having seroprotective antibody titers at D0, and the number participants was ~3.5x higher than UGA5, which had ~13% of participants in this category. The H3N2 components also induced the highest proportion participants in this category (~22%) compared to any of the other vaccine components of each vaccine across the 6 cohorts (**Fig 3C**). On average, ~83% of the participants entered each season seropositive to the B/Yamagata component of the vaccine and maintained that seropositivity following vaccination. The UGA1 cohort had the lowest percentage of these individuals (~67%) and the UGA5 cohort had the highest percentage (~90%). Of these participants, ~16% entered each season seropositive to the B/Yamagata component of the vaccine and had a 4-fold rise in titer following vaccination. In the UGA1 cohort, ~4 times as many participants (~33%) were in this category than in the UGA5 season (~8%) (**Fig 3C**). Across the UGA1-6 cohorts, the highest frequency of people (~87%) were seropositive on D0 and D28 to the B/Victoria component of the annual vaccine. The UGA5 cohort had the largest percentage of people (~90%) in this category and the UGA1 cohort had the fewest (~63%). Conversely, across the UGA1-6 cohorts, the B/Victoria components of the vaccine elicited HAI activity in the fewest number of participants (~12%) who were seropositive at D0 and had a 4-fold rise in titer following vaccination. The UGA1 cohort (~36%) had ~4.5 times more participants in this category than the UGA4 cohort (8%) (**Fig 3C**). There were also participants who were seropositive at D0 and seronegative at D28 following vaccination, across all the vaccine components each season, but never exceeded ~2% in any individual season (**Fig 3C**).

### Effects of prior vaccination on seroprotective and seroconversion HAI titers

Participants in the UGA4 cohort (2019–2020) were subdivided into categories based on whether the participant self-reported previous administration of influenza vaccination in either of the prior two seasons (**Table 4**). In general, participants not vaccinated in the prior 2 seasons had the highest rate of seroconversion to all 4 vaccine components in the 2019–2020 Fluzone® vaccine. In addition, these same participants also had the highest percentage of seronegative participants at D0, prior to vaccination (**Table 4**). On average, ~30% of the participants that were unvaccinated in the prior 2 seasons were seropositive to the H1N1 component of the vaccine at D0. In contrast, ~45% of the participants that received the influenza vaccine the prior two consecutive seasons were seropositive to the H1N1 component at D0. For participants not vaccinated in the previous 2 seasons, ~71% were seronegative to the H1N1 strain prior to vaccination compared to ~55% of participants vaccinated in the previous two seasons. Following vaccination, ~71% of the seronegative (D0) participants not vaccinated in the previous 2 seasons were combined seropositive or seroconverted post-vaccination, whereas ~35% of the participants vaccinated in the past 2 seasons converted from a seronegative state prior to vaccination to a combined seropositive or seroconverted post-vaccination. Fewer than 5% of participants, previously vaccinated, that were seropositive prior to vaccination had a 4-fold rise in HAI titer (seroconverted) post-vaccination (**Table 4**).

**Table 4:** Effects of prior vaccination on seroprotective and seroconversion HAI titers.

Component	Group	n-value	Total SN at D0	SN at D0 & D28	SN at D0, SP at D28	SN at D0, SC at D28	Total SP at D0	SP at D0, SN at D28	SP at D0 & D28	SP at D0, SC at D28
**H1N1**	No Vax 2 Years	54	70.4%	28.9%	2.6%	68.4%	29.6%	0.0%	68.8%	31.3%
	Vax 2+ Years	312	54.8%	64.9%	15.2%	19.9%	45.2%	0.0%	95.0%	5.0%
**H3N2**	No Vax 2 Years	54	92.6%	38.0%	2.0%	60.0%	7.4%	0.0%	50.0%	50.0%
	Vax 2+ Years	312	77.2%	47.3%	2.9%	49.8%	22.8%	1.4%	71.8%	26.8%
**YAM**	No Vax 2 Years	54	57.4%	3.2%	3.2%	93.5%	42.6%	0.0%	47.8%	52.2%
	Vax 2+ Years	312	33.3%	51.9%	24.0%	24.0%	66.7%	3.4%	90.9%	5.8%
**VIC**	No Vax 2 Years	54	66.7%	19.4%	2.8%	77.8%	33.3%	0.0%	77.8%	22.2%
	Vax 2+ Years	312	41.3%	49.6%	19.4%	31.0%	58.7%	2.7%	93.4%	3.8%

* No Vax 2 Years = no influenza vaccination received in the two preceding seasons (2017–2018 and 2018–2019)

On average, ~60% of participants not vaccinated in the prior 2 seasons, who were seronegative at D0, seroconverted to the H3N2 component of the vaccine, compared to ~46% of the participants that were vaccinated in prior seasons. Almost all participants (~93%) that were not vaccinated in the prior 2 seasons were seronegative to H3N2 component prior to vaccination, and ~77% of participants that were vaccinated in the prior two seasons were also seronegative at D0. Approximately 7% of participants that were not vaccinated in the prior 2 seasons were seropositive to the H3N2 component at D0, and of this population ~50% had a 4-fold rise in HAI titer post-vaccination. In contrast, ~23% of participants that were vaccinated in the prior 2 seasons were seropositive at D0, and of this population ~27% seroconverted post-vaccination.

Participants that were seronegative at D0 and not vaccinated in the prior 2 seasons, had the highest average seroconversion rate (~94%) to the B/Yamagata component of the vaccine. In contrast, ~24% of the participants that were seronegative at D0 and vaccinated in the prior 2 seasons seroconverted to the B/Yamagata component. Approximately 57% of the participants that were not vaccinated in the prior 2 seasons were seronegative prior to vaccination, whereas ~33% of the participants that were vaccinated in the prior two seasons were seronegative prior to vaccination (**Table 4**). For participants that were vaccinated in the prior 2 seasons and were seropositive prior to vaccination, ~6% had a 4-fold rise in HAI titer post-vaccination to the B/Yamagata component.

For participants not vaccinated in the past two seasons, ~67% of them were seronegative to B/Victoria component of the vaccine at D0, compared to ~41% of participants that were vaccinated in the previous two seasons (**Table 4**). Of the participants not vaccinated in the past two seasons that were seronegative prior to vaccination, ~78% seroconverted post-vaccination. In contrast, ~31% of participants that were vaccinated in the previous two seasons seroconverted from a seronegative state post-vaccination. Fewer than 4% of the participants, who were seropositive at D0 and vaccinated in the previous two seasons, had a 4-fold rise in HAI titers post-vaccination to the B/Victoria component, compared to ~22% of the participants who were not vaccinated in the previous 2 seasons (**Table 4**).

### Longitudinal tracking of seropositivity in UGA4 to UGA6

**Fig 4:**
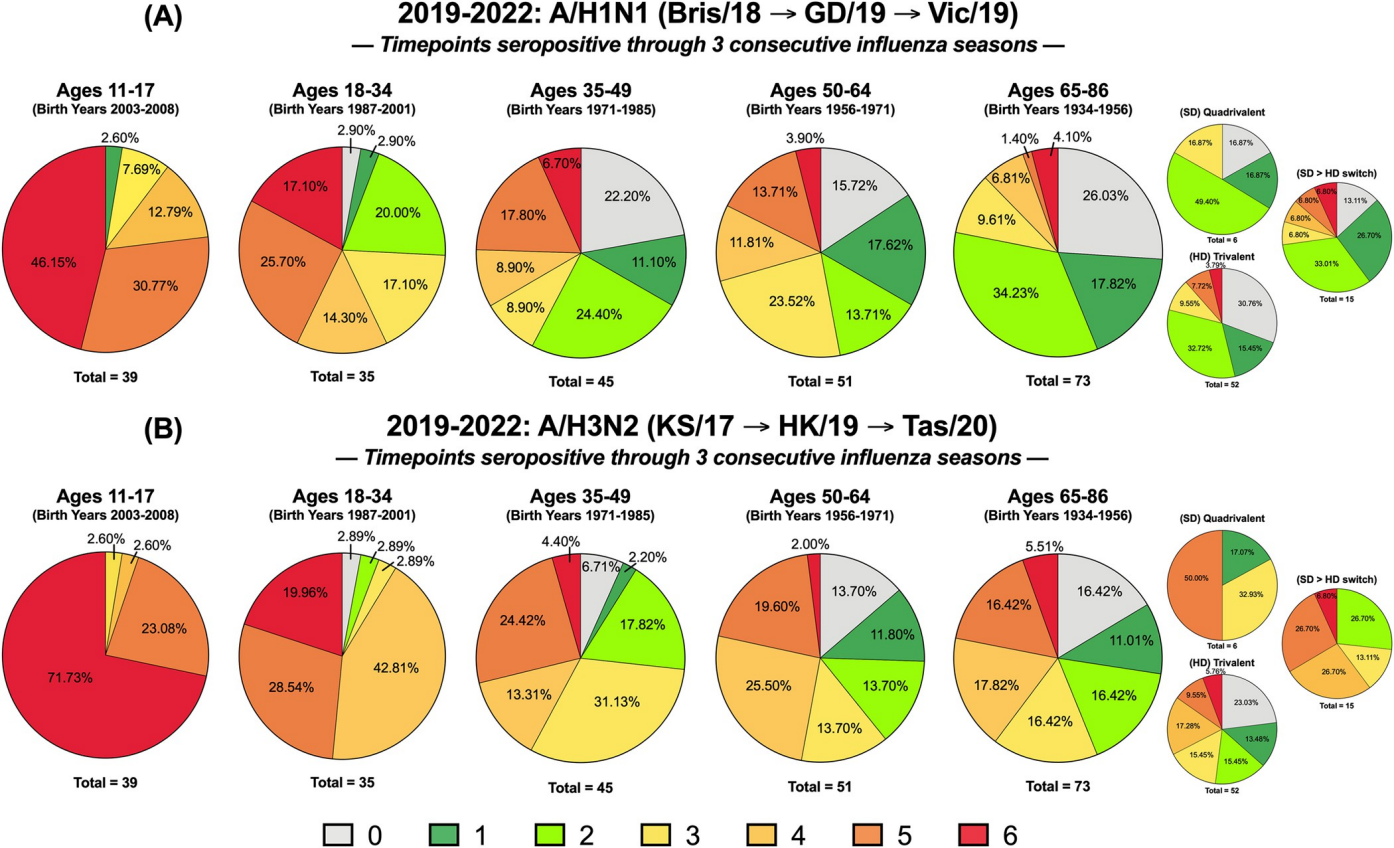
IAV seropositivity over multiple influenza seasons. Participants were immunized over 3 consecutive seasons, 2019–2020, 2020–2021, and 2021–2022 and were assessed for seropositive titers to the (A) H1N1 or (B) H3N2 components of the vaccine at day 0 and day 28. Immunized participants were categorized according to their seroprotective status at each of the 6 time points analyzed (*i*.*e*., days 0 and 28 in all 3 years). Subjects in each age group that had HAI titers less than 40 at all 6 time points were assigned a value of “0”. Subjects with an HAI titer of 40 or greater at one of the 6 time points was categorized as a “1”. Subjects with an HAI titer of 40 or greater at two of the 6 times was categorized as a “2”, at three of the 6 time points, was categorized as a “3”, at four of the 6 times was categorized as a “4”, at five of the 6 times was categorized as a “5”, and subjects with was greater than 40 all 6 times was categorized as a “6”. Pie charts for each of these 7 categories was assessed for each age group against the H1N1 or H3N2 component. The n value for each age group was listed beneath each pie chart. The 65–86 y.o. group is shown as a collective and further divided based on receivers of high dose (HD) trivalent vaccine versus the standard dose (SD) quadrivalent vaccine.

A longitudinal cohort of individuals that participated in the UGA4-6 studies every year from 2019–2022 was evaluated for HAI seropositivity on D0 and D28 of each year. Every timepoint where an individual had a seropositive antibody titer, they were assigned a score of “1”, and for every timepoint where their antibody titer was seronegative, they were assigned a score of “0”. Therefore, over the 3-year period, an individual could have a minimum seropositivity score of “0” and a maximum score of “6” (**Fig 4**). Participants aged 11–17 y.o. had the highest percentage of individuals (~46%) with a maximum seropositivity score of 6 against the H1N1 component of the vaccines across 2019–2022. However, this seropositivity rate was lower for H1N1 than against the H3N2, B/Yamagata, or B/Victoria components of the vaccine. This age group also had no people with a minimum seropositivity score of 0 (**Fig 4A**). In general, the percentage of individuals with maximum seropositivity scores to the H1N1 component of the vaccines decreased across the different groups as their age increased. Less than ~7% of people older than 35 y.o. had a seropositivity score of 6 to the H1N1 component of the vaccines (**Fig 4A**). Similarly, individuals aged 35 and above also had the highest percentage of participants (>16%) with a minimum seropositivity score of 0 across the 3 years (**Fig 4A**).

Participants aged 11–17 y.o. also had the highest percentage of seropositivity scores of 6 (~72%) to the H3N2 component of the vaccines across the UGA4-6 cohorts (**Fig 4B**). This proportion was ~4 times higher than the 18–34 y.o. age group and ~13 times higher than groups containing participants older than 35 y.o. (**Fig 4B**). Additionally, there were no people in the 11–17 y.o. group that had a seropositivity score less than 3. The 65–86 y.o. group had the highest proportion of participants with a minimum seropositivity score of 0 (~16%) and all these individuals received the IIV-HD vaccine each season (**Fig 4B**). There were ~3 times more people with seropositivity scores of 4 or above in this age group to the H3N2 component of the vaccines as there were to the H1N1 components (**Fig 4B**).

**Fig 5:**
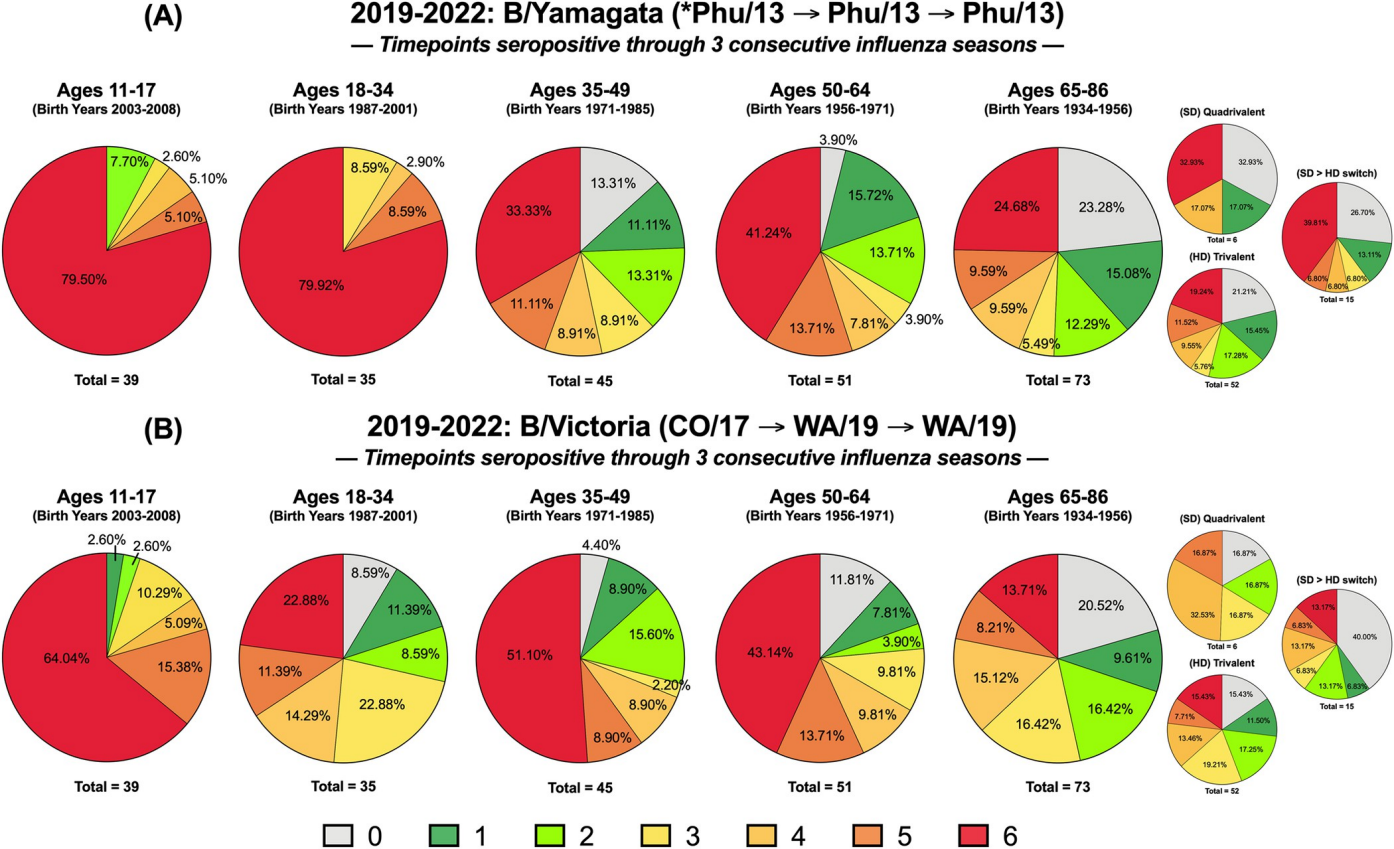
IBV seropositivity over multiple influenza seasons. Participants were immunized over 3 consecutive seasons, 2019–2020, 2020–2021, and 2021–2022 and were assessed for seropositive titers to the (A) Yamagata and (B) Victoria components of the vaccine at day 0 and day 28. Each volunteer was scored 0–6 according to their seroprotective status at each of the 6-time points analyzed (days 0 and 28 in all 3 years). Subjects in each age group that had HAI titers less than 40 at all 6-time points were assigned a value of “0”. Subjects with a HAI titer of 40 or greater at 1/6 time points were categorized as a “1” and so forth until “6” represents those that had a HAI titer of 40 or greater at 6/6 timepoints. The *n*-value for each age group is listed beneath each pie chart. The 65–86 y.o. group is shown as a collective and further divided based on receivers of high dose (HD) trivalent vaccine versus the standard dose (SD) quadrivalent vaccine.

The highest cumulative seropositivity scores across all the age groups was observed against the B/Yamagata component of the 2019–2022 vaccines (**Fig 5**). Individuals less than 35 y.o. had the highest seropositivity scores, and ~80% of the participants in this age group were seropositive at all 6 timepoints from 2019–2022 (**Fig 5A**). Over this period, there were ~50% as many participants aged 35 y.o. or older who had maximum seropositivity scores against the B/Yamagata component of the vaccines. The oldest group of individuals, aged 65–86 y.o., had the highest proportion of individuals (~23%) who did not have seropositive antibody titers at any of the 6 time points from 2019–2022. Participants in this age group vaccinated with the quadrivalent IIV-SD vaccine had the highest proportion of individuals (~33%) that were not seropositive at any of the time points analyzed (**Fig 5A**). Those who received the trivalent IIV-HD formulation every season were not immunized with the B/Yamagata component during the first season of analysis, 2019–2020.

The youngest individuals in the cohort, aged 11–17 y.o., also had the highest proportion of people who were seropositive at all 6 time points (~64%) compared to the other age groups against the B/Victoria components of the vaccines (**Fig 5B**). Participants aged 65–86 y.o. had the lowest percentage of individuals with a maximum seropositivity score (~14%) and the highest proportion of people with minimum seropositivity scores (~21%) (**Fig 5B**). There were no individuals aged 65–86 y.o. vaccinated with the quadrivalent IIV-SD vaccine that were seropositive at all 6 timepoints to the B/Victoria components of the vaccines. There were participants in this age group that had maximum seropositivity scores, but all these individuals received the IIV-HD vaccine at some point during the 2019–2022 seasons (**Fig 5B**).

## Discussion

In this study, 737 participants were enrolled, consented, and received commercial influenza vaccines over six consecutive influenza seasons (2016–2017 to 2021–2022). Blood was collected prior to vaccination and 3–5 timepoints post-vaccination. Sera, plasma, peripheral blood mononuclear cells (PBMC), as well as urine, RNA, and whole blood were collected in selected seasons. Influenza strains are updated annually, and during our 6-season study there were 5 different H1N1 strain changes, 5 different H3N2 strain changes, and 3 different B/Victoria strain changes. The B/Yamagata strain remained the same, but the emergence of SARS-CoV-2 at the end of 2019 seemingly eradicated the B/Yamagata-lineage [19, 20]. Overall, this dataset provides informative results regarding immune responses across different age groups before and after vaccination and analyzes immune responses between those vaccinated in previous seasons and those receiving a vaccination for the first time in 1 to 2 years.

The effectiveness of influenza vaccines can be influenced by multiple factors, including gender, race/ethnicity, age, health status and co-morbidities, as well as the host immune system. Children, pregnant women, and the elderly (≥65 years) are among the most susceptible populations to severe disease caused by the influenza virus. Individuals over the age of 65 are the fastest growing population and they have increased morbidity and mortality due to influenza virus infection, most commonly in combination with secondary bacterial and viral infections [21].

In this study, most participants vaccinated with split-inactivated influenza vaccine had a minimum of a 2-fold increase in anti-HA antibody titers within 4 weeks post-vaccination. Typically, older children and young adults had high, seroprotective HAI titers prior to vaccination each season that were maintained or then increased over time post-vaccination. However, the vaccine-elicited antibody titers waned over the subsequent year in all age groups, and declined more prominently in people 65 years of age or older, usually below a seroprotective titer (<40). As people age, they have reduced immune responses to new antigens. In the elderly, immunological changes occur that include impairments in immune activation and maintenance of immune memory cells [22, 23]. In the case of influenza virus infections, even though the hospitalization rates for adults over 70 years of age and children less than 5 years are almost identical, people ≥70 years old have increased occurrences of severe disease and death [24, 25]. Overall, influenza vaccination reduces hospitalization rates. However, protection induced by influenza vaccines in the elderly is reduced compared to the adults with impairments in both humoral and cellular immunity [26]. Additionally, cell-mediated immune responses to vaccinations are decreased in the elderly [27, 28]. In this study, influenza vaccination did not elicit high antibody titers in elderly participants, but the vaccine was effective at boosting low HAI titers to seroprotective levels. Gender differences also play a role, with females generally displaying higher antibody titers following vaccination than males [29, 30]. However, in this study, there was no observed statistical difference in HAI titers between male and female participants. Body Mass Index (BMI) also impacts immune responses elicited by influenza vaccines, with severely obese individuals having a reduced influenza vaccine induced immune response [31, 32]. However, in this study there was no correlation between BMI and reduced influenza vaccine seroconversion. The use of BMI as a measure of influenza vaccine effectiveness, and as a general health measure, is often controversial [33]. Despite decreased influenza vaccine induced antibody titers, people with high BMI are more likely to have severe influenza virus infections and illness compared to adults with healthy BMI levels [34]. Therefore, using antibody titers alone to determine vaccine effectiveness in obese or high BMI populations is challenging. Alternative measures such as serum leptin levels may more closely align with influenza vaccine seroconversion. Increased leptin titers are associated with impaired innate and adaptive immune functions including increased intrinsic B cell inflammation and reduced B cell function [35] and may be responsible for decreased production of protective antibodies in the elderly [36, 37].

In addition to the impact of age and pre-existing influenza immunity from past infections [38, 39], seasons with significant antigenic change between vaccine antigens can also impact antibody responses to influenza vaccines. An example was observed in the UGA4 cohort, where the new H3N2 vaccine strain selected for the 2019–2020 season was a clade 3C.3a.1 virus, KS/17 [40]. The KS/17 HA had the loss of two potential glycosylation sites (T128A and T160K) compared to the clade 3c.2a1, Sing/16, HA used in the previous season [41]. H3N2 HA molecules isolated from viruses in 2013–2016 belonging to clades 3C.2a or 3C.2a1 typically possess a serine at residue 144 in antigenic site A, which is in close proximity to the receptor binding domain [42]. This amino acid substitution removed a putative N-linked glycosylation site in H3N2 HA residues 144–146 that were present in earlier vaccine strains, including Switz/13 that also belongs to clade 3c.3a [41, 43]. These putative N-linked glycosylation sites may be a key driver in both the elicitation of antibodies with HAI activity to different epitopes on the HA protein. Additionally, these glycans can block the ability of pre-existing antibodies elicited by 3c.3a viruses to bind to the HA molecule by shielding specific epitopes on 3c.2a viruses. Overall, participants entering the 2019–2020 season did not have antibodies that efficiently recognized the H3N2 clades circulating and therefore, entered the season with low HAI titers to these new strains (**Fig 2**). However, vaccination with the 2019–2020 influenza vaccine elicited HAI activity in all age groups.

A similar phenomenon occurred in the UGA5 cohort, where antigenic changes in the H1N1 component resulted in participants entering the 2020–2021 season with low levels of pre-existing antibodies to the GD/19 antigen. The GD/19 virus is a member of clade 6B.1A.5a.1 and differs from the previous vaccine strain, Bris/18 (clade 6B.1A1), by 9 amino acids [44]. Most notably, 2 substitutions in antigenic site Sb, D187A and Q189E, increased the hydrophobicity of this epitope to facilitate viral escape from some neutralizing antibodies [45]. Antigenic site Sb is located on the distal tip of the of the HA monomer, near the sialic acid receptor binding domain, and alterations to this epitope can have large impacts on human HAI immunogenicity [46]. Overall, antigenic changes due to vaccine reformulation caused the serum HAI activity to be low at D0 against the vaccine strains used in UGA4 and UGA5 prior to vaccination, but following vaccination, titers increased on average for participants regardless of age.

The history of a participant’s immune history with the influenza virus can also significantly impact antibody responses to new influenza vaccines. Regardless of age, immunological recall or ‘back-boosting’ to antigenically related strains were associated with seroconversion to the vaccine strain(s) with imprinting exposure differing across age groups [47, 48]. Antibody cross-reactivity to past hemagglutinin antigenic variants may shape immune responses elicited by current split inactivated influenza vaccines. Younger individuals tend to have a more limited immunological history than the elderly. Therefore, their earlier influenza virus exposures are more closely related to currently circulating strains, resulting in an antibody repertoire that predominantly recognizes recently circulating viruses [38, 39]. Often, in young adults, neutralizing antibody titers remain high each season to circulating strains, unless there is an antigenic shift that introduces a new strain that younger people have not encountered, such as the H1N1 influenza virus that caused the 2009 pandemic that resulted in increased morbidity and mortality in people under 35 years of age [49, 50]. The addition or deletion of glycosylation sites in HA can also result in less effective antibodies, even in young adults, such as the deletion of the putative glycosylation sites in the KS/17 HA that was the H3N2 component in 2019–2020 season [51].

Participants that had not been vaccinated in the prior 2 years before vaccination in this study had a robust antibody response with higher rates of seroconversion and higher post-vaccination HAI titers, regardless of age, than participants that were vaccinated 2+ consecutive seasons. People vaccinated in multiple back-to-back seasons have been associated with lower vaccine effectiveness rates compared to people not vaccinated in the prior 5 seasons before vaccination [52, 53]. Similar results were also observed in people not vaccinated in the prior 2 seasons [54]. Overall, vaccine induced immune responses in participants vaccinated annually may result in lower vaccine effectiveness against viral infection, despite receiving annual vaccination. Participants in this study that were vaccinated in consecutive seasons had reduced serum HAI activity against the vaccine strains compared to participants that had not been vaccinated in the proceeding 2 years prior to entering this study. In the UGA4 cohort, newly vaccinated participants that were seronegative prior to vaccination were, on average, ~2X as likely to seroconvert to the 4 vaccine strains than participants consecutively vaccinated over the prior three seasons. However, individuals that received vaccines in the previous 2 seasons were more likely to be seropositive at D0 against all 4 vaccine antigens compared to participants who were not consecutively vaccinated. Participants that were not vaccinated in the 2 previous seasons were more likely to seroconvert at a higher rate than the annually vaccinated participants because their antibody titers were lower at D0 and had the capacity to increase from a lower baseline compared to participants that entered that season with higher antibody titers. These participants have more immunological space to observe rise in antibody titers, since the initial HAI titer is at a lower baseline than participants who are vaccinated every season. In addition, annual vaccination is necessary for older aged participant whose antibody titers tend to decline to non-protective levels from one year to the next. Seasonal vaccination might not raise antibody titers as significantly in populations that are vaccinated annually, however, annual vaccination is necessary for many individuals in order to boost antibody titers to protective levels, even if the titers are not maintained from season to season. Therefore, While the mechanism for these effects is not fully understood, annual vaccination may refocus antibodies to historical epitopes compared to contemporary epitopes resulting in cross-reactive, immune responses [55–57]. In addition, the antigenic distance hypothesis attempts to explain the variation in repeated vaccination by determining if vaccine effectiveness is influenced by the antigenic similarity between the prior season vaccine strain and a variant strain or the antigenic similarity between the current and the prior season’s vaccine strains [58]. This model shows that repeated vaccination can lead to higher or lower infection rates compared to first-time vaccinees depending upon the relatedness of the strains between two seasons, which is most likely related to their overall immune response to vaccination. While this study did not focus on vaccine effectiveness to prevent viral infection, participants in this study that were not vaccinated in the preceding seasons had more robust HAI antibodies post-vaccination than participants vaccinated each season that supports these hypotheses.

In order to fully understand the host influences on vaccination and the ever-changing influenza variants over time, samples from these cohorts have been analyzed for host RNA gene expression profiles by transcriptomics [59], host glycomics [60], proteomics [61], and metabolomics [62], as well as DNA methylation and immune senescence [63]. These data sets have been used to develop predictive models of host responses and vaccine effectiveness [64]. Overall, multiple factors contribute to the elicitation of influenza vaccine induced immune responses, including, among others, the age, health status, pre-immune immune history to influenza, as well as the antigenic shift and drift of influenza strains. We expect that the public availability of these datasets will provide influenza virus researchers opportunities to uncover additional insights and mechanisms of vaccine induced immunity responses. Taken together, this rich set of longitudinal results from participants in this influenza vaccine research study can provide a clearer picture of human responses to influenza virus vaccination.

## Supporting information

S1 Fig HAI activity in serum antibody elicited by Fluzone^®^ during the 2016–2017 season for the UGA1 cohort. HAI titers against the four strains included in the 2016–2017 Fluzone^®^ influenza vaccine are plotted in a box-and-whisker plot for each age group comparing pre-vaccination (D0) and post-vaccination (D21) titers. The box covers 50% of all values, with the lower (Q1) and upper (Q4) quartiles shown for the box ends, and the median value as a dividing line. The whiskers extend to the lowest and highest titers. A two-tailed paired Student t-test with Wilcoxon-sign rank test is used to compare vaccine-induced titer changes (*p≤0.05; **p≤0.01; ***p≤0.001; ****p≤0.0001). The n-value per age group is listed on the x-axis. Note: Fluzone^**®**^ high-dose was a trivalent formulation this season with no B/Yamagata component, so many participants aged 65 and older were not immunized by it; however, post-vaccination titers are still recorded.
(TIF)

S2 Fig HAI activity in serum antibody elicited by Fluzone^®^ during the 2017–2018 season for the UGA2 cohort. HAI titers against the four strains included in the 2017–2018 Fluzone^®^ influenza vaccine are plotted in a box-and-whisker plot for each age group comparing pre-vaccination (D0) and post-vaccination (D21) titers. The box covers 50% of all values, with the lower (Q1) and upper (Q4) quartiles shown for the box ends, and the median value as a dividing line. The whiskers extend to the lowest and highest titers. A two-tailed paired Student t-test with Wilcoxon-sign rank test is used to compare vaccine-induced titer changes (*p≤0.05; **p≤0.01; ***p≤0.001; ****p≤0.0001). The n-value per age group is listed on the x-axis. Note: Fluzone^**®**^ high-dose was a trivalent formulation this season with no B/Yamagata component, so many participants aged 65 and older were not immunized by it; however, post-vaccination titers are still recorded.
(TIF)

S3 Fig HAI activity in serum antibody elicited by Fluzone^®^ during the 2018–2019 season for the UGA3 cohort. HAI titers against the four strains included in the 2018–2019 Fluzone^®^ influenza vaccine are plotted in a box-and-whisker plot for each age group comparing pre-vaccination (D0) and post-vaccination (D21) titers. The box covers 50% of all values, with the lower (Q1) and upper (Q4) quartiles shown for the box ends, and the median value as a dividing line. The whiskers extend to the lowest and highest titers. A two-tailed paired Student t-test with Wilcoxon-sign rank test is used to compare vaccine-induced titer changes (*p≤0.05; **p≤0.01; ***p≤0.001; ****p≤0.0001). The n-value per age group is listed on the x-axis. Note: Fluzone^**®**^ high-dose was a trivalent formulation this season with no B/Yamagata component, so many participants aged 65 and older were not immunized by it; however, post-vaccination titers are still recorded.
(TIF)

S4 Fig HAI activity in serum antibody elicited by Fluzone^®^ during the 2019–2020 season for the UGA4 cohort. HAI titers against the four strains included in the 2019–2020 Fluzone^®^ influenza vaccine are plotted in a box-and-whisker plot for each age group comparing pre-vaccination (D0) and post-vaccination (D21) titers. The box covers 50% of all values, with the lower (Q1) and upper (Q4) quartiles shown for the box ends, and the median value as a dividing line. The whiskers extend to the lowest and highest titers. A two-tailed paired Student t-test with Wilcoxon-sign rank test is used to compare vaccine-induced titer changes (*p≤0.05; **p≤0.01; ***p≤0.001; ****p≤0.0001). The n-value per age group is listed on the x-axis. Note: Fluzone^**®**^ high-dose was a trivalent formulation this season with no B/Yamagata component, so many participants aged 65 and older were not immunized by it; however, post-vaccination titers are still recorded.
(TIF)

S5 Fig HAI activity in serum antibody elicited by Fluzone^®^ during the 2020–2021 season for the UGA5 cohort. HAI titers against the four strains included in the 2020–2021 Fluzone^®^ influenza vaccine are plotted in a box-and-whisker plot for each age group comparing pre-vaccination (D0) and post-vaccination (D21) titers. The box covers 50% of all values, with the lower (Q1) and upper (Q4) quartiles shown for the box ends, and the median value as a dividing line. The whiskers extend to the lowest and highest titers. A two-tailed paired Student t-test with Wilcoxon-sign rank test is used to compare vaccine-induced titer changes (*p≤0.05; **p≤0.01; ***p≤0.001; ****p≤0.0001). The n-value per age group is listed on the x-axis.
(TIF)

S6 Fig HAI activity in serum antibody elicited by Fluzone^®^ during the 2021–2022 season for the UGA6 cohort. HAI titers against the four strains included in the 2020–2021 Fluzone^®^ influenza vaccine are plotted in a box-and-whisker plot for each age group comparing pre-vaccination (D0) and post-vaccination (D21) titers. The box covers 50% of all values, with the lower (Q1) and upper (Q4) quartiles shown for the box ends, and the median value as a dividing line. The whiskers extend to the lowest and highest titers. A two-tailed paired Student t-test with Wilcoxon-sign rank test is used to compare vaccine-induced titer changes (*p≤0.05; **p≤0.01; ***p≤0.001; ****p≤0.0001). The n-value per age group is listed on the x-axis.
(TIF)

S1 File HAI raw data file. Excel file containing all HAI data for UGA1 through UGA6 for all viruses included in this publication and more. Each season separated as a sheet or tab within the file.
(XLSX)

S2 File Data analysis file used for Figs 2 and 3 and Table 4. Excel file containing details on how the HAI data was analyzed for Figs 2 and 3 (data transposed to PRISM to design figures) and Table 4.
(XLSX)

S3 File Data analysis file used for Figs 4 and 5. Excel file containing details on how the HAI data was analyzed for Figs 4 and 5 (data transposed to PRISM to design figures).
(XLSX)

S4 File ELISA raw data file. Excel file containing ELISA data for UGA1 through UGA6. IgG and IgA data for most serum samples, but many incomplete. Each season separated as a sheet or tab within the file. The coating antigens matched each of the four vaccine strains for most seasons, but sometimes the previously included vaccine component was used, as noted for each season.
(XLSX)
