# Supplementary figures and images for: Longitudinal assessment of human antibody binding to hemagglutinin elicited by split-inactivated influenza vaccination over six consecutive seasons
Source: PLoS One. 2024 Jun 25;19(6):e0301157. doi: 10.1371/journal.pone.0301157 (PMC11198804; doi:10.1371/journal.pone.0301157)

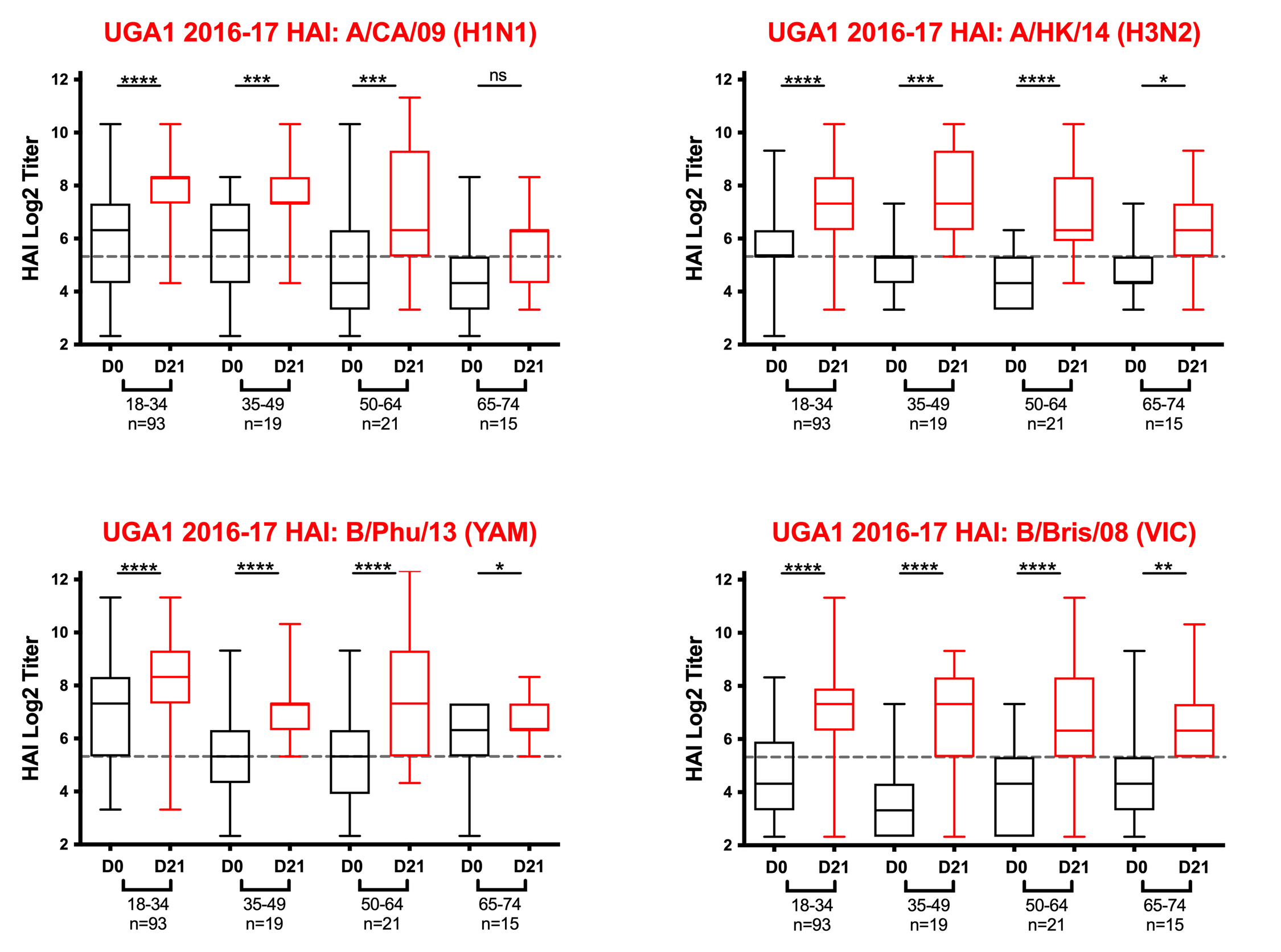

Supplement: S1 Fig — HAI titers against the four strains included in the 2016–2017 Fluzone® influenza vaccine are plotted in a box-and-whisker plot for each age group comparing pre-vaccination (D0) and post-vaccination (D21) titers. The box covers 50% of all values, with the lower (Q1) and upper (Q4) quartiles shown for the box ends, and the median value as a dividing line. The whiskers extend to the lowest and highest titers. A two-tailed paired Student t-test with Wilcoxon-sign rank test is used to compare vaccine-induced titer changes (*p≤0.05; **p≤0.01; ***p≤0.001; ****p≤0.0001). The n-value per age group is listed on the x-axis. Note: Fluzone® high-dose was a trivalent formulation this season with no B/Yamagata component, so many participants aged 65 and older were not immunized by it; however, post-vaccination titers are still recorded. (TIF) [file pone.0301157.s001.tif]

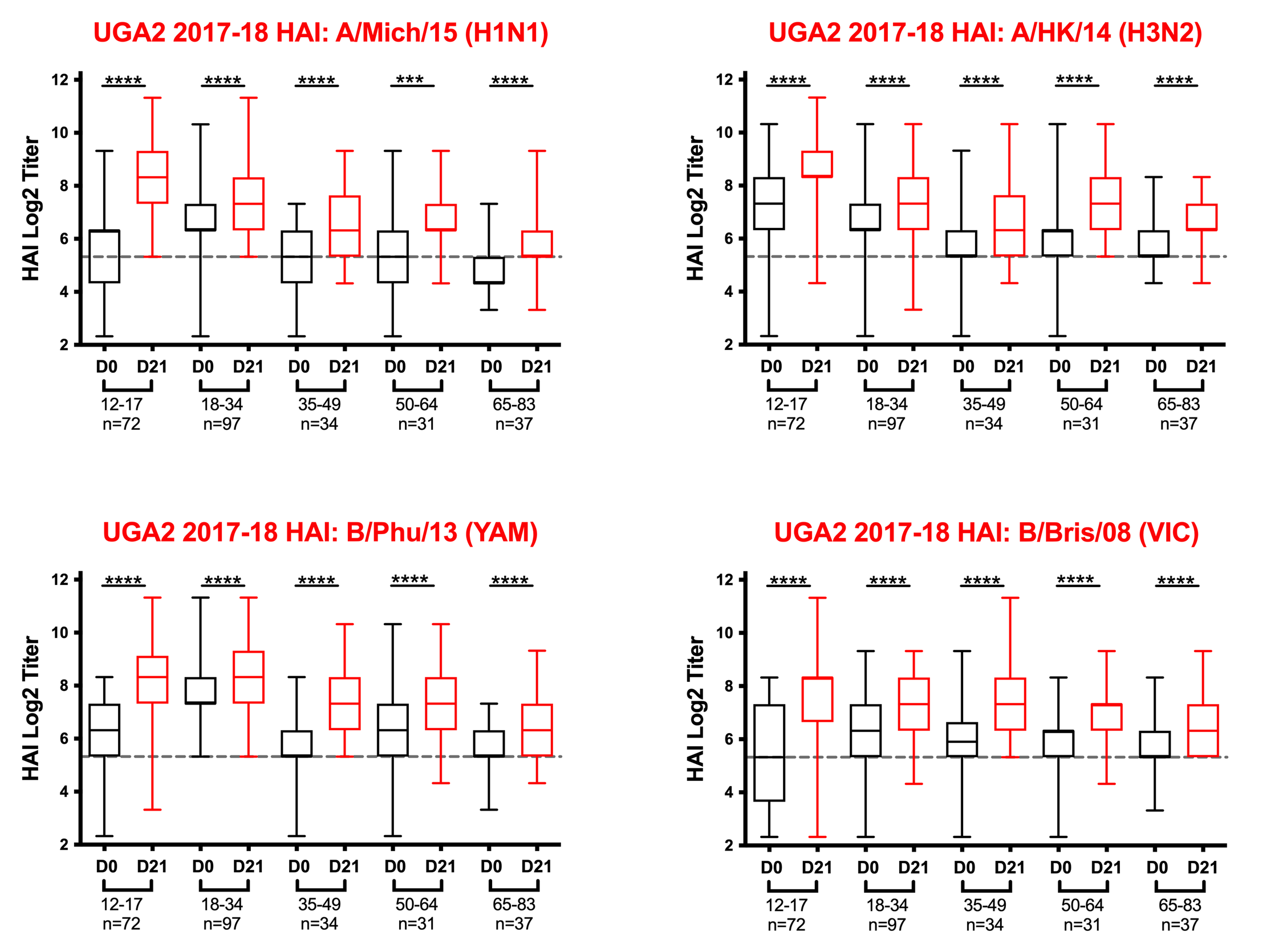

Supplement: S2 Fig — HAI titers against the four strains included in the 2017–2018 Fluzone® influenza vaccine are plotted in a box-and-whisker plot for each age group comparing pre-vaccination (D0) and post-vaccination (D21) titers. The box covers 50% of all values, with the lower (Q1) and upper (Q4) quartiles shown for the box ends, and the median value as a dividing line. The whiskers extend to the lowest and highest titers. A two-tailed paired Student t-test with Wilcoxon-sign rank test is used to compare vaccine-induced titer changes (*p≤0.05; **p≤0.01; ***p≤0.001; ****p≤0.0001). The n-value per age group is listed on the x-axis. Note: Fluzone® high-dose was a trivalent formulation this season with no B/Yamagata component, so many participants aged 65 and older were not immunized by it; however, post-vaccination titers are still recorded. (TIF) [file pone.0301157.s002.tif]

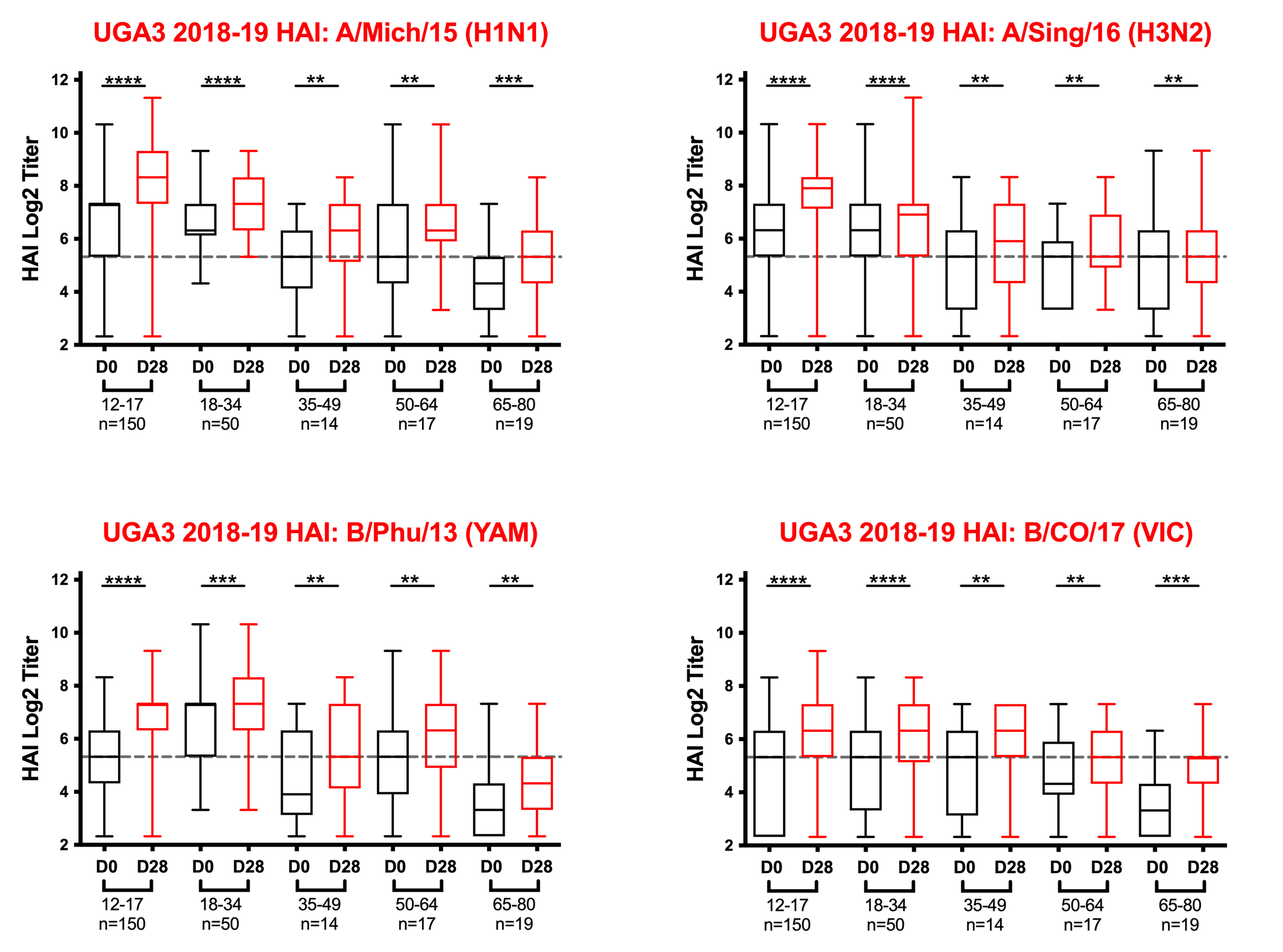

Supplement: S3 Fig — HAI titers against the four strains included in the 2018–2019 Fluzone® influenza vaccine are plotted in a box-and-whisker plot for each age group comparing pre-vaccination (D0) and post-vaccination (D21) titers. The box covers 50% of all values, with the lower (Q1) and upper (Q4) quartiles shown for the box ends, and the median value as a dividing line. The whiskers extend to the lowest and highest titers. A two-tailed paired Student t-test with Wilcoxon-sign rank test is used to compare vaccine-induced titer changes (*p≤0.05; **p≤0.01; ***p≤0.001; ****p≤0.0001). The n-value per age group is listed on the x-axis. Note: Fluzone® high-dose was a trivalent formulation this season with no B/Yamagata component, so many participants aged 65 and older were not immunized by it; however, post-vaccination titers are still recorded. (TIF) [file pone.0301157.s003.tif]

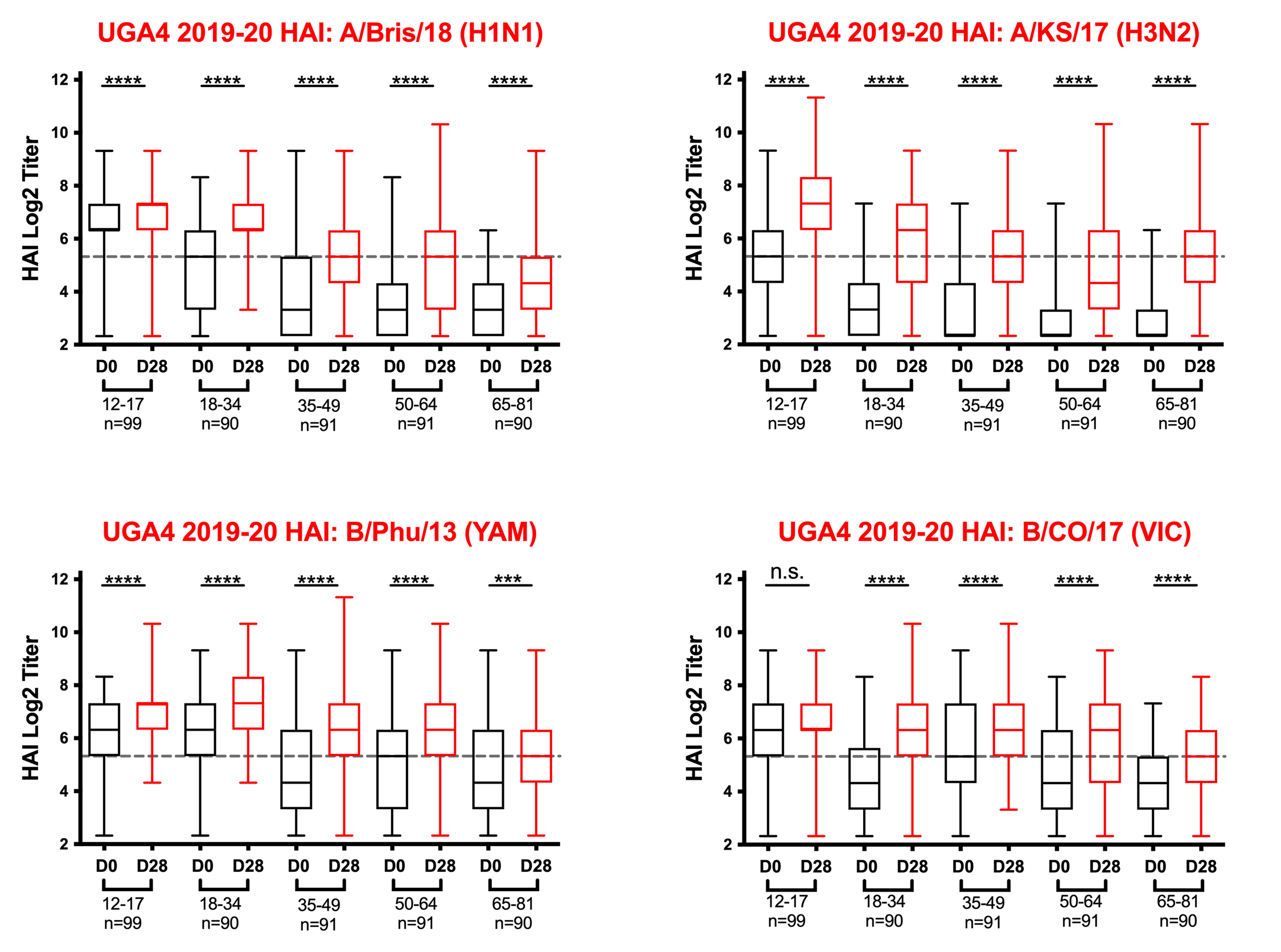

Supplement: S4 Fig — HAI titers against the four strains included in the 2019–2020 Fluzone® influenza vaccine are plotted in a box-and-whisker plot for each age group comparing pre-vaccination (D0) and post-vaccination (D21) titers. The box covers 50% of all values, with the lower (Q1) and upper (Q4) quartiles shown for the box ends, and the median value as a dividing line. The whiskers extend to the lowest and highest titers. A two-tailed paired Student t-test with Wilcoxon-sign rank test is used to compare vaccine-induced titer changes (*p≤0.05; **p≤0.01; ***p≤0.001; ****p≤0.0001). The n-value per age group is listed on the x-axis. Note: Fluzone® high-dose was a trivalent formulation this season with no B/Yamagata component, so many participants aged 65 and older were not immunized by it; however, post-vaccination titers are still recorded. (TIF) [file pone.0301157.s004.tif]

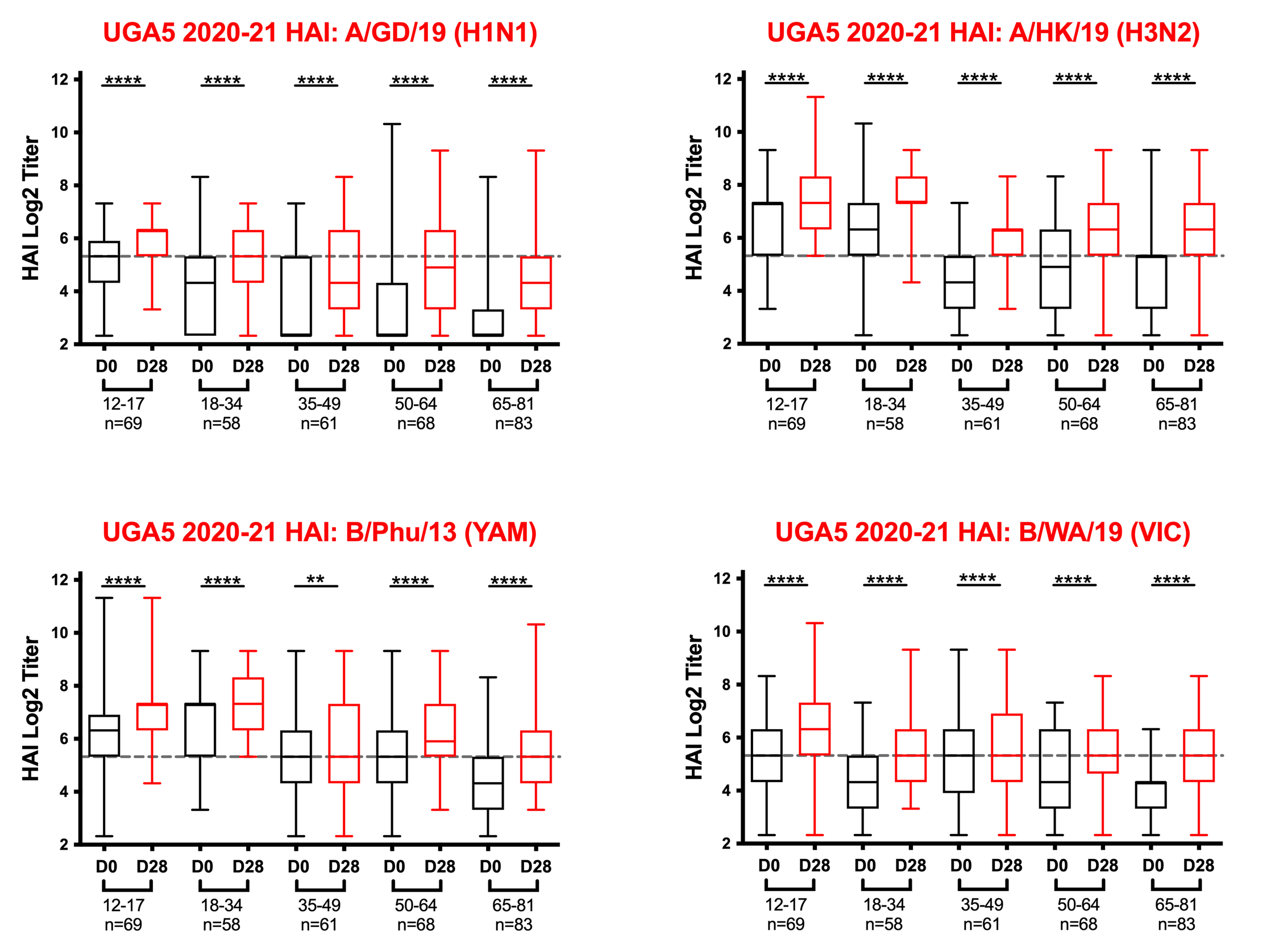

Supplement: S5 Fig — HAI titers against the four strains included in the 2020–2021 Fluzone® influenza vaccine are plotted in a box-and-whisker plot for each age group comparing pre-vaccination (D0) and post-vaccination (D21) titers. The box covers 50% of all values, with the lower (Q1) and upper (Q4) quartiles shown for the box ends, and the median value as a dividing line. The whiskers extend to the lowest and highest titers. A two-tailed paired Student t-test with Wilcoxon-sign rank test is used to compare vaccine-induced titer changes (*p≤0.05; **p≤0.01; ***p≤0.001; ****p≤0.0001). The n-value per age group is listed on the x-axis. (TIF) [file pone.0301157.s005.tif]

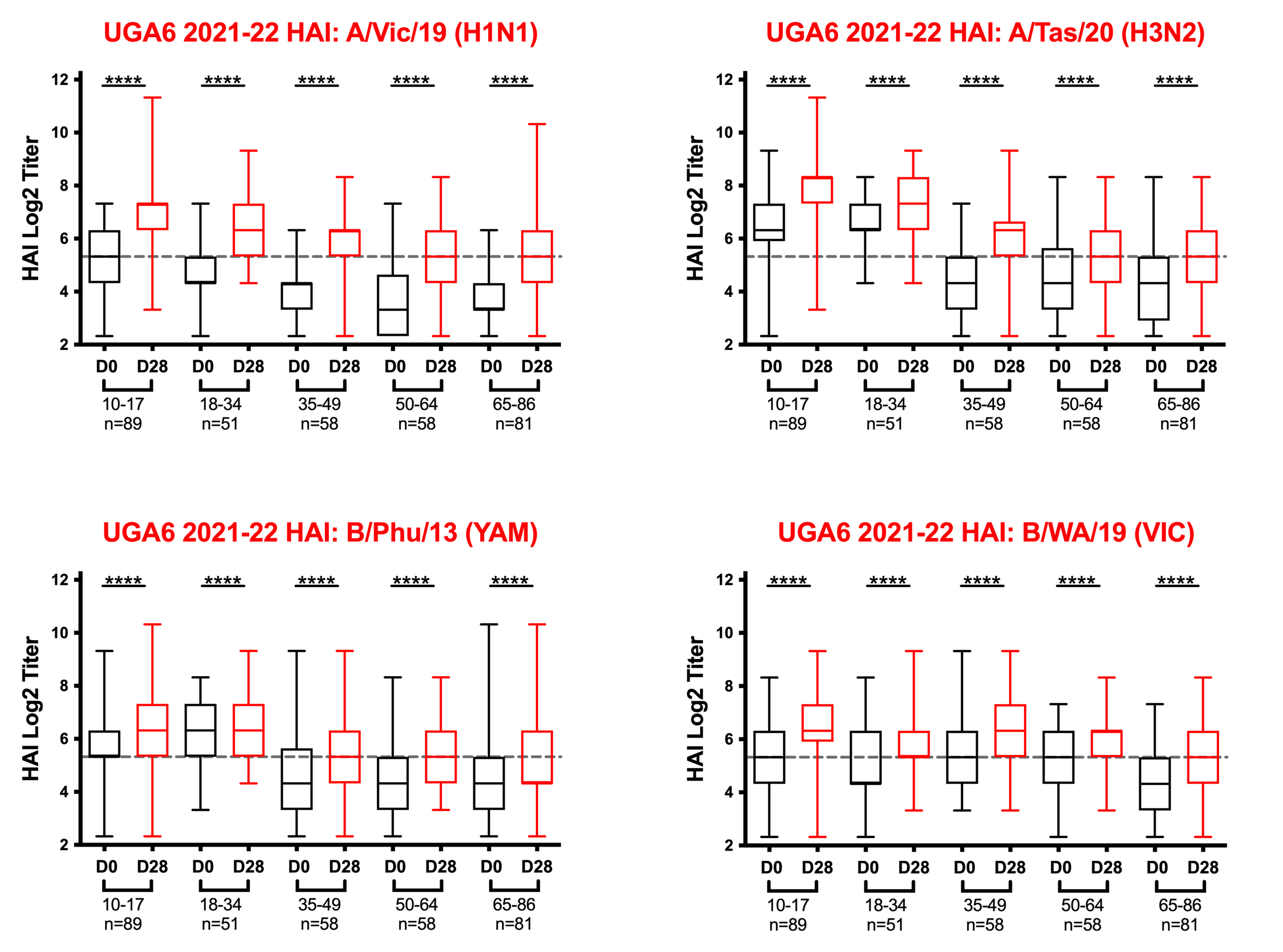

Supplement: S6 Fig — HAI titers against the four strains included in the 2020–2021 Fluzone® influenza vaccine are plotted in a box-and-whisker plot for each age group comparing pre-vaccination (D0) and post-vaccination (D21) titers. The box covers 50% of all values, with the lower (Q1) and upper (Q4) quartiles shown for the box ends, and the median value as a dividing line. The whiskers extend to the lowest and highest titers. A two-tailed paired Student t-test with Wilcoxon-sign rank test is used to compare vaccine-induced titer changes (*p≤0.05; **p≤0.01; ***p≤0.001; ****p≤0.0001). The n-value per age group is listed on the x-axis. (TIF) [file pone.0301157.s006.tif]
